# Stepwise De‐solvation and Diffusion Kinetics of Hydrated Zn‐ion in Hierarchical Porous Carbon Anode for Improved EDLC Behavior

**DOI:** 10.1002/advs.202519684

**Published:** 2026-02-03

**Authors:** Qiang Qu, Zhi‐Zhen Chi, Zhi‐Wen Wang, Jia‐Qing Xie, Ling Qiu, Fang Gu, Ming‐Qiang Zhu

**Affiliations:** ^1^ College of Mechanical and Electronic Engineering Northwest Agriculture & Forestry University Yangling China; ^2^ College of Forestry Northwest Agriculture & Forestry University Yangling China; ^3^ Key Laboratory of Eucommia of National Forestry and Grassland Administration Yangling China; ^4^ Key Laboratory of Shaanxi Province on Development and Utilization of Economic Plant Resources Yangling China

**Keywords:** dimension compatibility, hydrated Zn‐ion, porous carbon anode, stepwise de‐solvation

## Abstract

Porous carbon (PC) is widely recognized as a promising anode material for zinc‐ion hybrid supercapacitors (ZiHSCs), but its practical deployment is hindered by sluggish ion diffusion kinetics and poor rate performance. In this study, a stepwise de‐solvation of hydrated Zn‐ion is observed during migration within the hierarchical micropore channels characterized by dominated dimension of 0.74 and 1.54 nm. This phenomenon mitigates the free energy dissipation of hydrated Zn‐ion diffusion, accelerates charge transfer kinetics, and substantially enhances the EDLC generation. In situ Raman, ex situ FT‐IR and XPS analysis reveal an intensive removal of bound water from [Zn (H_2_O)_6_]^2+^ and rapid micropore filling at the discharge state. The optimized anode delivers a specific capacitance of 224.1 mAh/g at 0.2 A/g, an impressive energy density of 179.6 Wh/kg (active materials basis), and exceptional cycling stability (99.1% capacity retention over 100,000 cycles). This dimension design paradigm establishes a generalizable framework for optimizing porous carbons in energy storage, bridging the gap between fundamental ion‐solvent‐pore interactions.

## Graphical abstract

The size mismatch between the micropore channel and [Zn(H_2_O)_6_]^2+^ in porous carbon anode during the discharge process was addressed by regulating the hierarchical channel. The inducted stepwise de‐solvation of [Zn(H_2_O)_6_]^2+^ significantly reduced its de‐solvation energy, promoted the EDLC behavior, and strengthened the diffusion kinetic.

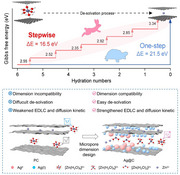

## 1 Introduction

Zn‐ion hybrid supercapacitors (ZiHSCs) attract significant attention due to their high power density, exceptional cycling stability (> 10^5^ cycles), and inherent safety advantages [1, 2]. Porous carbon (PC) anodes are a vital component in achieving this performance, possessing desirable attributes like simple preparation, abundant active sites, and structural stability [3]. However, practical ZiHSCs performance remains limited by sluggish Zn‐ion diffusion kinetics and poor rate performance [3, 4]. During operation, Zn‐ions dissolve from the zinc foil anode upon discharging [5, 6], subsequently migrating through the aqueous electrolyte as solvated [Zn (H_2_O)_6_]^2+^ complexes (average diameter of 0.86 nm) [7, 8]. Critically, the electrochemical double‐layer capacitive (EDLC) mechanism governing discharge storage in PC anodes requires complete de‐solvation of these [Zn (H_2_O)_6_]^2+^ complexes followed by adsorption of naked Zn‐ions into the ultra‐micropore channel (< 0.70 nm) [9]. This de‐solvation process presents a major kinetic bottleneck, as evidenced by the rate constant (k_0_ ≈ 10^−5^–10^−6^ cm/s) being orders of magnitude lower than typical electron transfer rates (k_0_ ≈ 10^−3^ cm/s) [10]. Consequently, this sluggish de‐solvation weakens the EDLC contribution and directly contributes to the rate performance degradation. Therefore, the primary challenge resides in optimizing the diffusion channel and enhancing the transfer kinetics of hydrated Zn‐ions within PC anodes to overcome these limitations.

Prior strategies of optimizing PC anodes to facilitate [Zn (H_2_O)_6_]^2+^ storage mainly focus on micropore size regulation, gradient pore construction, and heteroatom doping (e.g., N/P co‐doping) [11]. For instance, expanding mesopore distribution (2.0–20 nm) alleviates diffusion limitations imposed by ultra‐micropores (< 0.7 nm) [7]. Empirically, a PC anode with a dominant micropore (0.82 nm) close to [Zn (H_2_O)_6_]^2+^ delivers a high energy density of 158 Wh/kg in ZiHSC system [12]. Computational studies further reveal that the reduction of micropores raises the free energy barrier to [Zn (H_2_O)_6_]^2+^ diffusion, significantly hindering the ion diffusion kinetics [13]. However, these approaches neglect the spatial confinement effects when micropore diameters fall below that of [Zn (H_2_O)_6_]^2+^. This effect may lead to the stepwise de‐solvation of the solvated Zn^2+^ within the micropore channels, thereby promoting the entrance of naked Zn‐ion and rapid generation of EDLC. However, a comprehensive investigation into the interplay between optimized dimensions of pore channel, stepwise de‐solvation of [Zn (H_2_O)_6_]^2+^, and improved EDLC kinetics remains unexplored.

To systematically investigate the effect of micropore channel size on diffusion kinetic and rate performance of PC anode, a dimension‐tailored carbon composite (Ag@C) was synthesized through a simple solution adsorption method. The constricted micropores (dimensions of ∼0.74 and ∼1.54 nm) accelerated ion diffusion kinetic and strengthened EDLC generation. Importantly, the integration of experimental characterizations with density functional theory (DFT) calculations revealed that the stepwise de‐solvation behavior reduced the free energy dissipation of [Zn (H_2_O)_6_]^2+^ de‐solvation and promoted the adsorption of naked Zn^2+^ into the pore channel. As a result, the Ag@C anode achieved a high specific capacity of 224.1 mAh/g at 0.2 A/g, an energy density of 179.6 Wh/kg (active material basis), and 99.1% capacity retention over 100000 cycles. These properties surpass conventional PC anodes (175.3 mAh/g, 121.5 Wh/kg, 79.4% retention). By systematically optimizing micropore architecture and EDLC kinetics, this strategy paves the way for the commercialization of advanced ZiHSC.

## 2 Results and Discussion

### 2.1 The Structural Characteristic of PC and Modified Carbon Material

**FIGURE 1:**
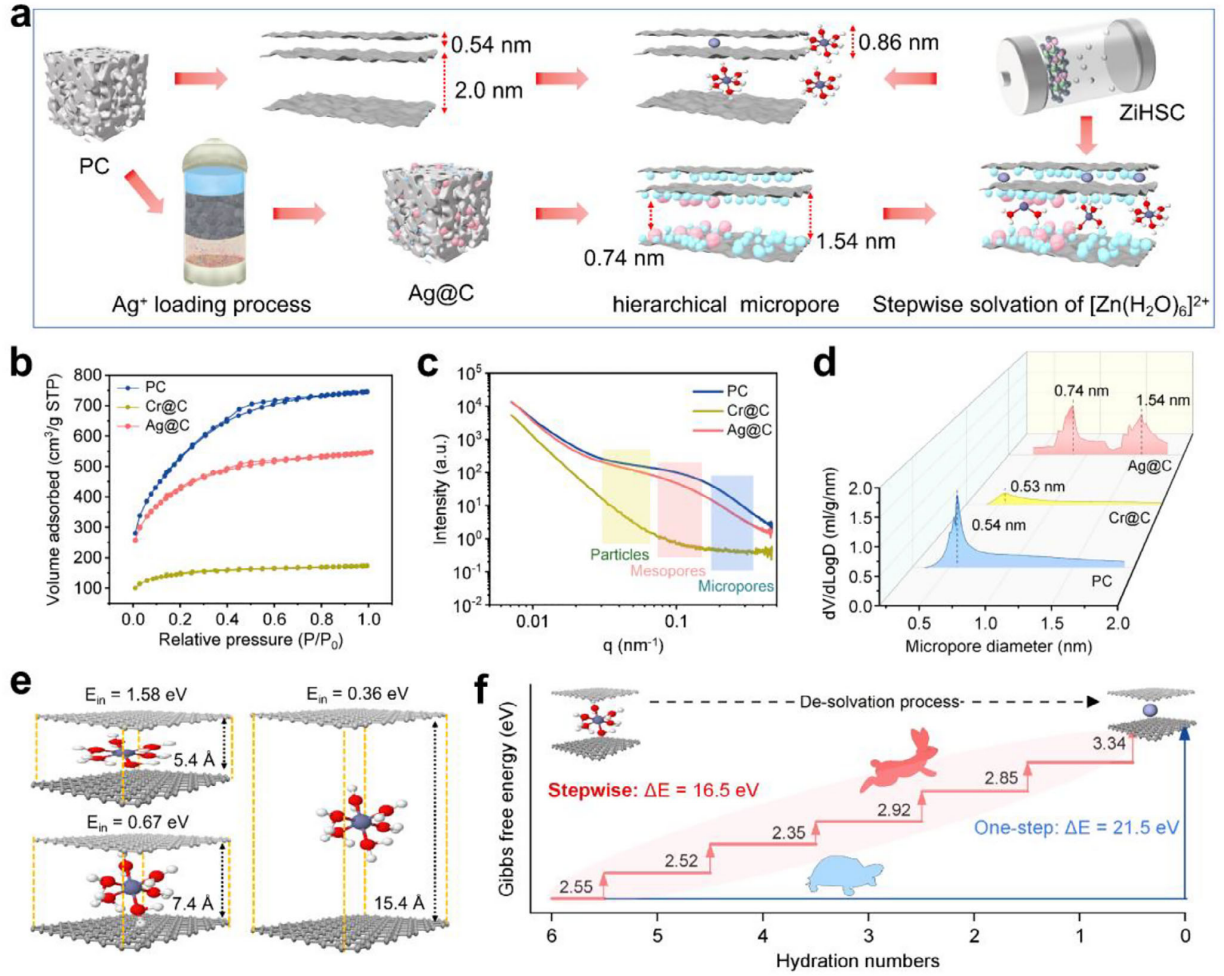
The schematic illustration of the synthesis of Ag@C sample (a), N_2_ adsorption–desorption isotherms of samples (b), SAXS spectra of samples (c), micropore diameter distribution of samples (d), schematic representation of [Zn (H_2_O)_6_]^2+^ diffused in the slit pores with a size of 5.4, 7.4 and 15.4 Å and the corresponding E_in_ calculated by DFT (e), de‐solvation energy of hydrated Zn‐ion in PC electrode by one step and Ag@C electrode by multiple steps (f).

The modified carbon anodes were synthesized through a simple adsorption of transition metal ions (Cr (VI)) and non‐transition metal ions (Ag (I)) in a solution system (Figure 1a). Cr (VI) and Ag (I) loadings were 11.21 and 1.13 wt.%, respectively, calculated by accounting for the concentration of the corresponding residual solution (Equations S1 and S2). Furthermore, the ion coordination was determined via *Langmuir* fitting isotherm (R^2^ = 0.9996/0.9976 vs *Freundlich* R^2^ = 0.9898/0.9958) (Figure S1 and Table S1) [3], indicating the ion coverage on pore channel surface and complexation with oxygen‐containing functional groups (OFGs) [14]. This ion coverage on the pore channel was further observed by Scanning Electron Microscopy (SEM) and Transmission Electron Microscope (TEM) techniques (Figures S2 and S3). Typically, the morphology of the PC product displayed an abundant pore structure [9]. After the ion‐coordination process, chromium‐containing particles filled the pores of Cr@C. In contrast, the distinct distribution of diamond‐shaped particles containing silver, attributed to the existence of Ag^0^, was observed on the pore channel of Ag@C [10]. TEM image and corresponding element mapping validated the uniformly anchored Ag specie. The high‐resolution TEM (HRTEM) images of Ag@C further revealed the lattice fringe of Ag specie with an interplanar spacing of 0.234 nm, corresponding to the (111) plane of Ag nanoparticles (NPs) (PDF#87‐0720) [6]. The decreased intensity of the amorphous carbon diffraction peak at around 26.54° on X‐Ray Diffraction (XRD) patterns indicated the reduced porosity of Cr@C and Ag@C (Figure S3) [15].

The starting PC sample exhibited classical type‐I/IV N_2_ sorption isotherms (Figure 1b) and characteristics of micropore‐dominated structures with mesopore contributions (specific surface area: 1402.8 m^2^/g) (Table 1) [16]. After the ion‐coordination process, the low‐pressure adsorption volume (P/P_0_ < 0.2) on the N_2_ sorption isotherms decreased, while the moderate‐pressure hysteresis loop (P/P_0_ = 0.2–0.6) gradually vanished. This variation indicated the micropore filling and mesopore channel constriction in both Cr@C and Ag@C (Figure S4) [17]. Furthermore, modified carbon samples (Cr@C/Ag@C) showed reduced SSA (490.6 and 959.8 m^2^/g), but increased average pore size (from 2.05 to 2.19 nm for Cr@C; 2.15 nm for Ag@C, respectively). This phenomenon was consistent with the reduced intensity of micropore and mesopore on Small Angle X‐ray Scattering (SAXS) patterns (Figure 1c and Figure S6) [18]. Moreover, PC sample displayed the typical ultra‐micropores with the dominated diameter of 0.54 nm, which would hinder the transfer of solvated [Zn (H_2_O)_6_]^2+^ (0.86 nm diameter) (Figure 1d). In contrast, the micropore structure in Ag@C gradually transformed into the sub‐nanometric channels (diameter of 0.74 and 1.54 nm). According to the analysis of SEM and TEM, this structure involution was resulted from the blockage of Ag (I) in ultra‐micropores and the Ag° filling on mesopore channel surface [18]. Consequently, the dominated micropore diameter of 0.74 nm may trigger the spatial confinement effect on [Zn (H_2_O)_6_]^2+^, leading to the partial water molecular loss and stepwise de‐solvation of hydrated Zn‐ion.

**TABLE 1:** Porous properties of PC and modified carbon samples.

Samples	S_BET_ ^a^	S_micro_ ^b^	S_meso_ ^c^	V_total_ ^d^	V_micro_ ^e^	V_meso_ ^f^	D_total_ ^g^	P_meso_ ^h^
	(m^2^/g)	(m^2^/g)	(m^2^/g)	(cm^3^/g)	(cm^3^/g)	(cm^3^/g)	(nm)	(%)
PC	1402.8	424.7	978.2	0.73	0.31	0.42	2.05	57.5%
Cr@C	490.6	199.7	290.9	0.27	0.12	0.15	2.19	54.2%
Ag@C	959.8	404.4	555.4	0.52	0.21	0.27	2.15	57.9%

^a^ Specific surface area was calculated in P/P_0_ pressure range from 0.05 to 0.30.

^b^ Micropore surface area was calculated from *t*‐plot method.

^c^ Mesopore surface area was calculated from *t*‐plot method.

^d^ Total pore volume at P/P_0_ = 0.95.

^e^ Micropore volume was calculated from *t*‐plot method.

^f^ V_meso_ = V_total_ ‐V_micro_.

^g^ Average pore diameter of total pore.

^h^ Proportion of mesoporous.

Based on the first principle, density functional theory (DFT) calculations of interaction energy (E_in_) within a bilayer graphene slit‐pore model could elucidate the effect of micropore dimension on the energy barrier for [Zn (H_2_O)_6_]^2+^ diffusion (Figure 1e) [8]. The high E_in_ (1.58 eV) of [Zn (H_2_O)_6_]^2+^ in the graphene layers with 0.54 nm interspace necessitated complete de‐solvation for ion access to active sites. But the slow de‐solvation reaction may present a kinetic bottleneck, delaying the rapid EDLC formation. In contrast, expanding bilayer graphene interface (1.54 nm) reduced E_in_ by 77% (0.36 eV), enabling direct diffusion of solvated Zn^2+^ without significant energy penalties. The sub‐nanometric pores (0.74 nm) also provided “open‐access” pathways, decreasing E_in_ to 0.67 eV. This may trigger slight de‐solvation and further optimize the solvent form of migrating [Zn (H_2_O)_x_]^2+^ (0<x<6). Furthermore, the energy loss of Zn^2+^ de‐solvation in different ways was quantified by graphene‐layered computational model (Figure 1f). Migration of [Zn (H_2_O)_6_]^2+^ from 2 to 0.56 nm ultra‐micropore in the pristine micropore channel required an extreme 21.52 eV barrier via one‐step de‐solvation. In contrast, within a hierarchical micropore structure (sequentially 2, 1.54, and 0.74 nm), spatial confinement could achieve stepwise de‐solvation reaction of [Zn (H_2_O)_6_]^2+^ prior to ultra‐micropore entry. The reduced energy loss (16.53 eV) revealed a critical advantage of stepwise de‐solvation in hierarchical micropores. It drastically reduced energy penalties compared to one‐step de‐solvation, which could accelerate hydrated Zn‐ion diffusion kinetic and promote the EDLC generation.

Raman spectroscopy revealed distinct structural evolution in Cr@C and Ag@C, showing reduced disorder ratio (*A*_D_/*A*_G_ = 2.396 and 2.289) compared to that of PC (*A*_D_/*A*_G_ = 2.836) (Table S2 and Figure S7). This tendency indicated that ion coordination and metal complex pore‐filling diminished the defect region [18]. Furthermore, the formation of metal complexes in both Cr@C and Ag@C (Figures S8 and S9) was confirmed by the enhanced intensity of the C═O/C─O peak on FT‐IR spectra and the Cr/Ag element diffraction peak on the XPS spectra of the total survey [19, 20]. Among the XPS spectra, the enhanced relative contents of C─O and C═O group on the C 1s and O 1s spectra of Cr@C and Ag@C suggested the formation of C─O─M complexes (M = metal) (Figure S10 and S11) [21]. Meanwhile, the Cr 2p spectra were deconvoluted into the Cr 2p_1/2_ and Cr 2p_3/2_ peaks (Figure S12). The distribution ratio of Cr (III) (83.7%) and Cr (VI) (16.2%) indicated that chromium‐containing compounds could provide electrons for Zn^2+^ stripping/deposition [22]. Ag 3d spectra of Ag@C confirmed the Ag^0^ presence (matching the SEM observation), which may alter the charge distribution during charge and discharge, and distinctly enhanced the conductivity (101.9 S/m vs PC 0.5 S/m, Figure S13). The improvement of water contact angel (WCA) for Ag@C demonstrated the strengthened hydrophilic behavior after ion‐coordination process (Figure S14), indicating the enhanced anode wettability in aqueous electrolyte.

### 2.2 The Zn‐ion Diffusion Kinetics Characteristics of PC and Modified Porous Carbon

**FIGURE 2:**
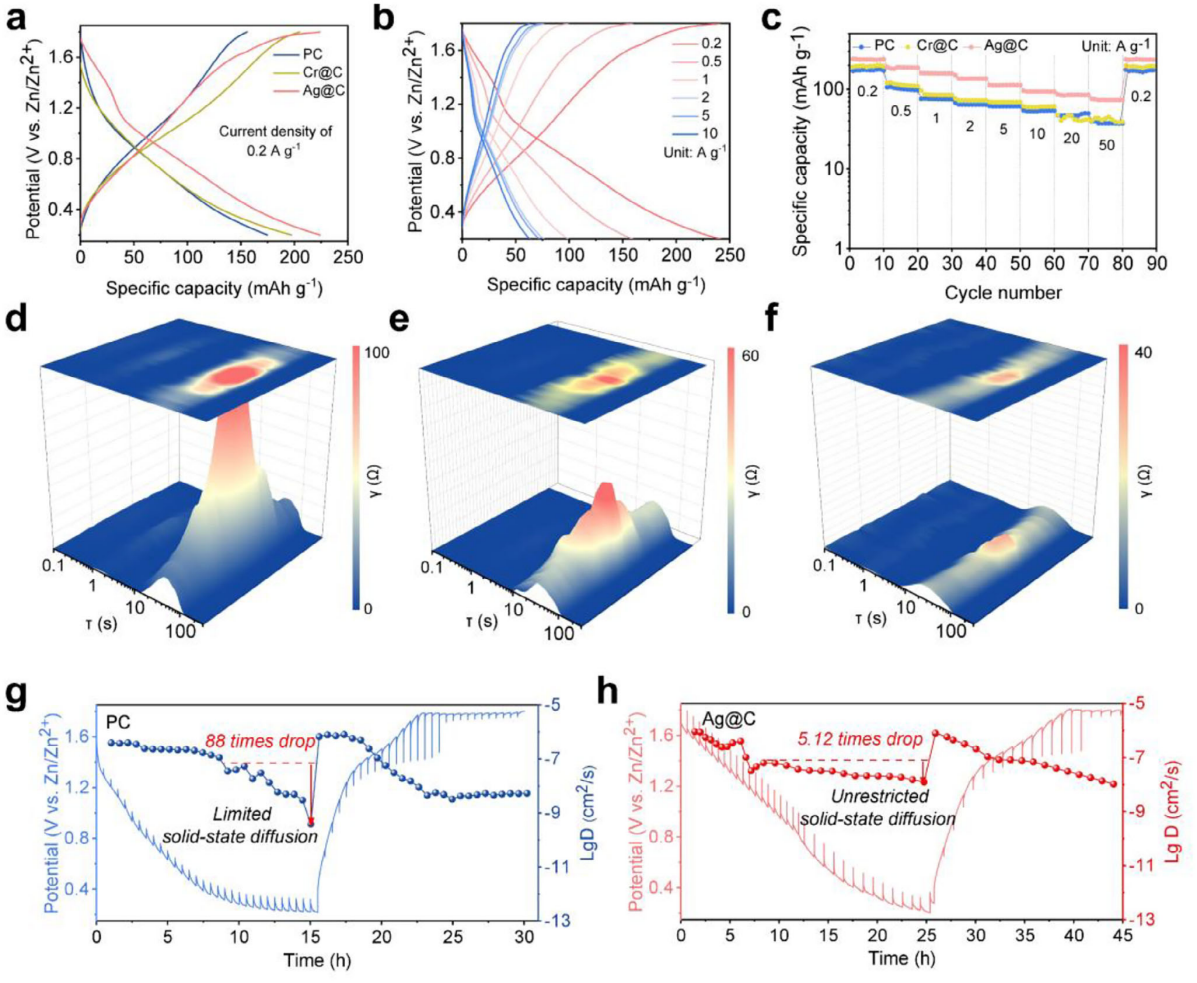
The discharge and charge curves of PC and modified carbon electrode (a), discharge and charge curves of Ag@C at different current density (b), rating performance of PC and modified carbon anode (c), DRT plots of PC anode (d), DRT plots of Cr@C anode (e), DRT plots of Ag@C anode (f), GITT curves of PC anode (g) and Ag@C anode (h).

To evaluate the functional impact of micropore channel modification on Zn^2+^ storage, a ZiHSC device was assembled by using carbon anodes paired with Zn foil anode. Compared to the specific capacity of PC anode (175.3 mAh/g at 0.2 A/g), modified carbon anodes demonstrated the superior capacity of 197.4 mAh/g (Cr@C) and 224.1 mAh/g (Ag@C) (Figure 2a). Notably, Ag@C anode retained 31.7% of initial capacity at ultrahigh current density (100 A/g), which exceeded the rate performance of both PC (20.0%) and Cr@C (6.2%) (Figure 2b,c and Figure S15). This superiority of the Ag@C electrode demonstrated the strengthened EDLC behavior under the condition of rapid electron transfer. This enhancement could be attributed to the reduced diffusion energy barrier of hydrated Zn‐ions within the expanded micropore channels.

To correlate micropore channel dimension with charge transport kinetic, Galvanostatic Electrochemical Impedance Spectroscopy (GEIS) was collected, and the EIS spectra of all anodes at 0.2 V discharge state were fitted using Z‐View software to obtain an equivalent circuit diagram (Figures S16 and S17, and Table S5). The EIS spectrum of Ag@C anode exhibited a lower high‐frequency semicircle (*R*ct = 23.2 Ω) that that of Cr@C (35.7 Ω) and PC anode (50.5 Ω), respectively. The *R*ct reduction of Ag@C anode reflected the improved kinetic for charge transfer [23]. Furthermore, the EIS data during the discharge and charge process were fitted by in situ Distribution Relaxation Times (DRT) modeling. For the starting PC, the mounted intensity of τ_5_ peak (τ = 0.1–1 s) indicated the slow charge transfer rate, likely due to the size mismatch between the micropore channel and the hydrated Zn‐ion (Figure 2d). Meanwhile, the broad shape and high intensity of the τ_7_ peak (τ = 10–100 s) indicated the significant polarization phenomenon during Zn ion diffusion process [24]. This could be attributed to that the intensive one‐step de‐solvation of [Zn (H_2_O)_6_]^2+^ delayed the entry of naked Zn ions into ultra‐micropore channels [25]. In contrast, the reduced intensity of the τ_5_ peak for Cr@C and Ag@C anode suggested the strengthened Zn ion transfer rate. This phenomenon elucidated the enhanced pseudocapacitance by the formed transmission layer of loaded chromium compound and sliver specie (Figure 2e,f). Furthermore, the diminished intensity of τ_7_ peak observed for Ag@C anode demonstrated the alleviative polarization [2]. This advantage stemmed from the strengthened Zn ion diffusion by stepwise [Zn (H_2_O)_6_]^2+^ de‐solvation in expanded micropore channels.

Galvanostatic Intermittent Titration Technique (GITT) measurements were conducted to reveal the evolution of the Zn^2+^ diffusion coefficient (D_Zn_^2+^) during discharge/charge processes [24]. The starting PC anode displayed a D_Zn_^2+^ of about 10^−7^ cm^2^/s until the potential was discharged to 1.4 V, after which it decreased to approximately 1.14% of this initial value in the low potential region (Figure 2g). This inhibited diffusion kinetic indicated the significant polarization due to the slow one‐step de‐solvation reaction of [Zn (H_2_O)_6_]^2+^ [25]. In contrast, the D_Zn_^2+^ of Ag@C anode declined as discharge processed to 1.4 V, which was caused by an early stepwise de‐solvation reaction of [Zn (H_2_O)_6_]^2+^ within sub‐nano micropore channels (Figure 2h). Notably, the obvious reduction of polarization at low potentials associated with the D_Zn_^2+^ of Ag@C anode remained 19.53% of the initial level [24].

**FIGURE 3:**
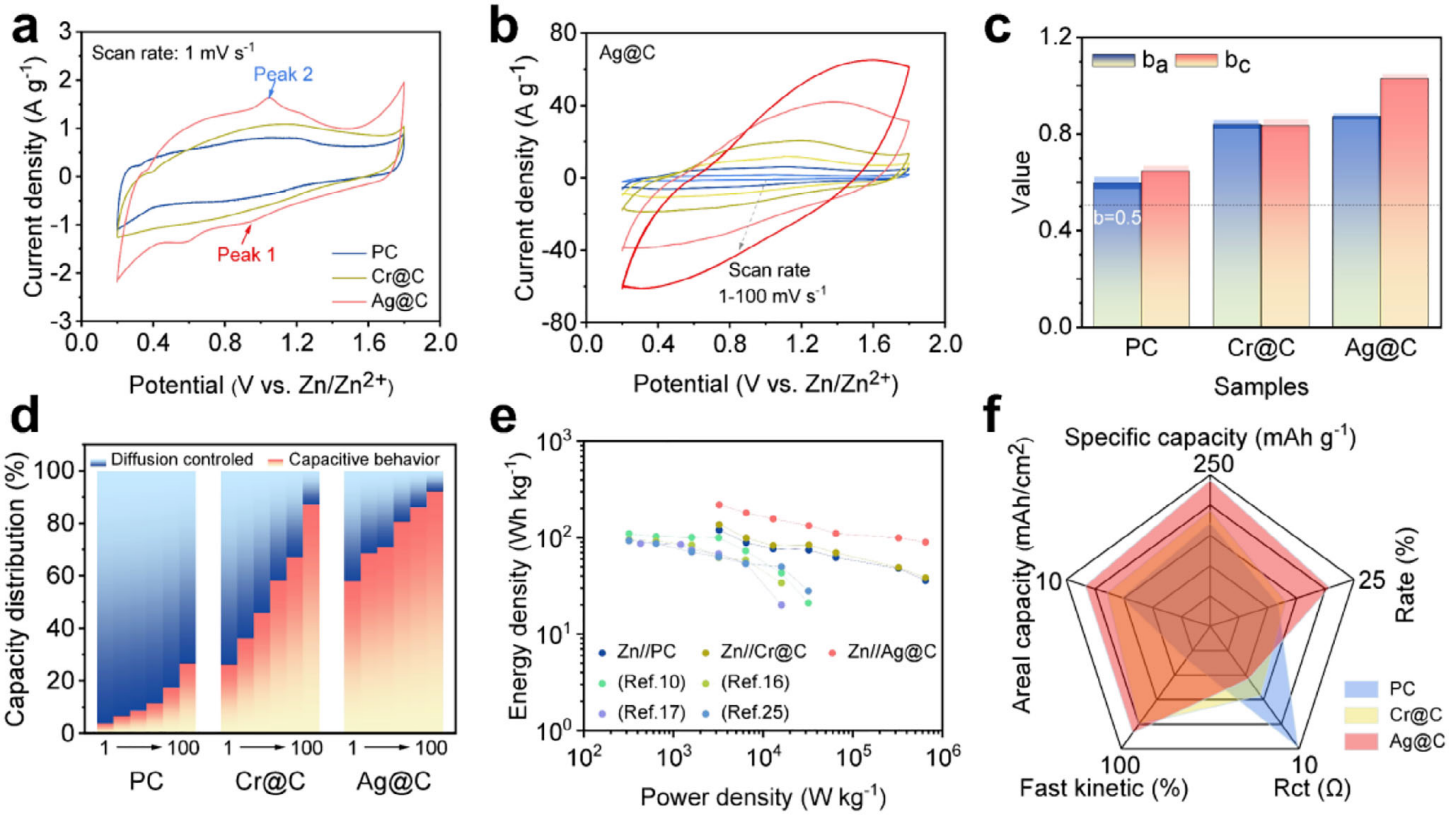
The CV curves of PC and modified carbon electrodes at 1 mV/s scan rate (a), CV curves of Ag@C electrode at different scan rate (b), linear relationships between logarithm currents and logarithm sweep rate of PC and modified carbon electrodes (c), diffusion controlled and capacitive controlled distribution ratio of PC and modified carbon electrodes at different scan rate (d), Ragone plot of electrodes in this work compared with previous researches (e), and radar plot for five properties of PC and modified carbon electrodes (f).

Cyclic Voltammetry (CV) curves recorded at 1 mV/s exhibited the distinct redox peaks (E_pa_ ≈ 1.21 V, E_pc_ ≈ 0.95 V) (vs Standard Calomel Electrode), which were likely attributed to stripping/deposition reaction of Zn/Zn^2+^ (Figure 3a) [26]. Furthermore, the increased current density of the redox peak observed for Ag@C suggested the augmented electron transfer and Zn‐ion diffusion during the charge/discharge process. As the scan rate increased from 0.1 to 100 mV/s, the redox peak currents for both PC and modified carbon anodes gradually declined, indicating a transition from faradaic pseudocapacitance to EDLC dominance (Figure 3b and Figure S18).

To investigate charge‐storage dynamics, CV data was analyzed using the power‐law relationship voltammograms peak current (*i*) and scanning rates (*v*) as follows,

(1) $$i=av^{b}$$

In general, the parameter b value of 0.5 could be attributed to sluggish reaction kinetics by diffusion behavior of Zn^2+^, while b value of 1.0 indicated ultrarapid reaction kinetic dominated by capacitive behavior [4]. PC anode exhibited b_a_ of 0.595 ± 0.025 and b_c_ of 0.644 ± 0.023, which suggested the electrochemical behavior was dominated by charge diffusion (Figure 3c and Figure S19). This sluggish kinetics was owing to the slow de‐solvation and restricted transfer rate of [Zn (H_2_O)_6_]^2+^ within narrow micropore channels. The calculated *b*‐value near 0.75 of the Cr@C electrode was assigned to a mixed mechanism of diffusion and capacitive behavior. In contrast, the distinct capacitance‐dominance behavior (b ≈ 1.0) of Ag@C demonstrated the amplified EDLC and faradaic pseudocapacitance formation within expanded micropore channels [27].

Besides, the *Dunn* method was employed to determine the relative contribution of ultrarapid capacitive‐controlled reaction and the sluggish diffusion‐controlled reaction [27]. The detailed calculation process was shown as the following equation (Equation 2),

(2) $$i\left(V\right)=i_{\mathrm{cap}}+i_{\mathrm{diff}}=k_{1}v+k_{2}v^{1/2}$$

where k_1_ and k_2_ were constants, k_1_ *v* represented current ascribed by ultrarapid capacitive behavior, and *i* was the total current. The diffusion‐controlled contribution rate to charge storage in the PC anode ranged from 3.8% to 61.9% at 1–100 mV/s scan rates (Figure 3d and Figure S20). In contrast, modified carbon anodes exhibited an enhanced capacitive dominance. In detail, Cr@C achieved 26.2% capacitive contribution at 1 mV/s scan rate, while Ag@C only exhibited 58.0% capacitive dominance rate (Figure 3d and Figures S21 and S22). Furthermore, this trend was intensified at elevated scan rates (5–100 mV/s), where Ag@C surged to a higher capacitive contribution of 92.6% than that of PC (26.6%). This superiority suggested that the expanded micropore channels enhanced spatial confinement on [Zn (H_2_O)_6_]^2+^, which induced the stepwise [Zn (H_2_O)_6_]^2+^ de‐solvation and promoted the EDLC generation. Additionally, the excellent areal capacity of Ag@C anode with an increased mass loading (2–32 mg/cm^2^) also revealed the fact that hierarchical pore channels can enhance the transport and storage of electrolyte ions in thick electrodes (Figure S23). Energy and power densities of all anodes were calculated using the gravimetric capacitance values derived from galvanostatic charge–discharge profiles (Equations S8 and S9). The PC anode delivered a modest energy density of 121.5 Wh/kg at a power density of 3240 W/kg (Figure 3e). By contrast, the Cr@C and Ag@C electrodes achieved the enhanced energy densities of 142.2 Wh/kg and 179.6 Wh/kg at the same power density. Up to now, the energy density outperformed the state‐of‐the‐art carbon‐based ZiHSC in reported papers. In summary, compared to pristine porous carbon anodes, Ag@C electrodes operated through stepwise de‐solvation of [Zn (H_2_O)_6_]^2+^ exhibited significant advantages in both Zn‐ions transfer kinetic and storage (Figure 3f).

### 2.3 The Mechanism of Promoted Performance in of PC and Modified Porous Carbon

**FIGURE 4:**
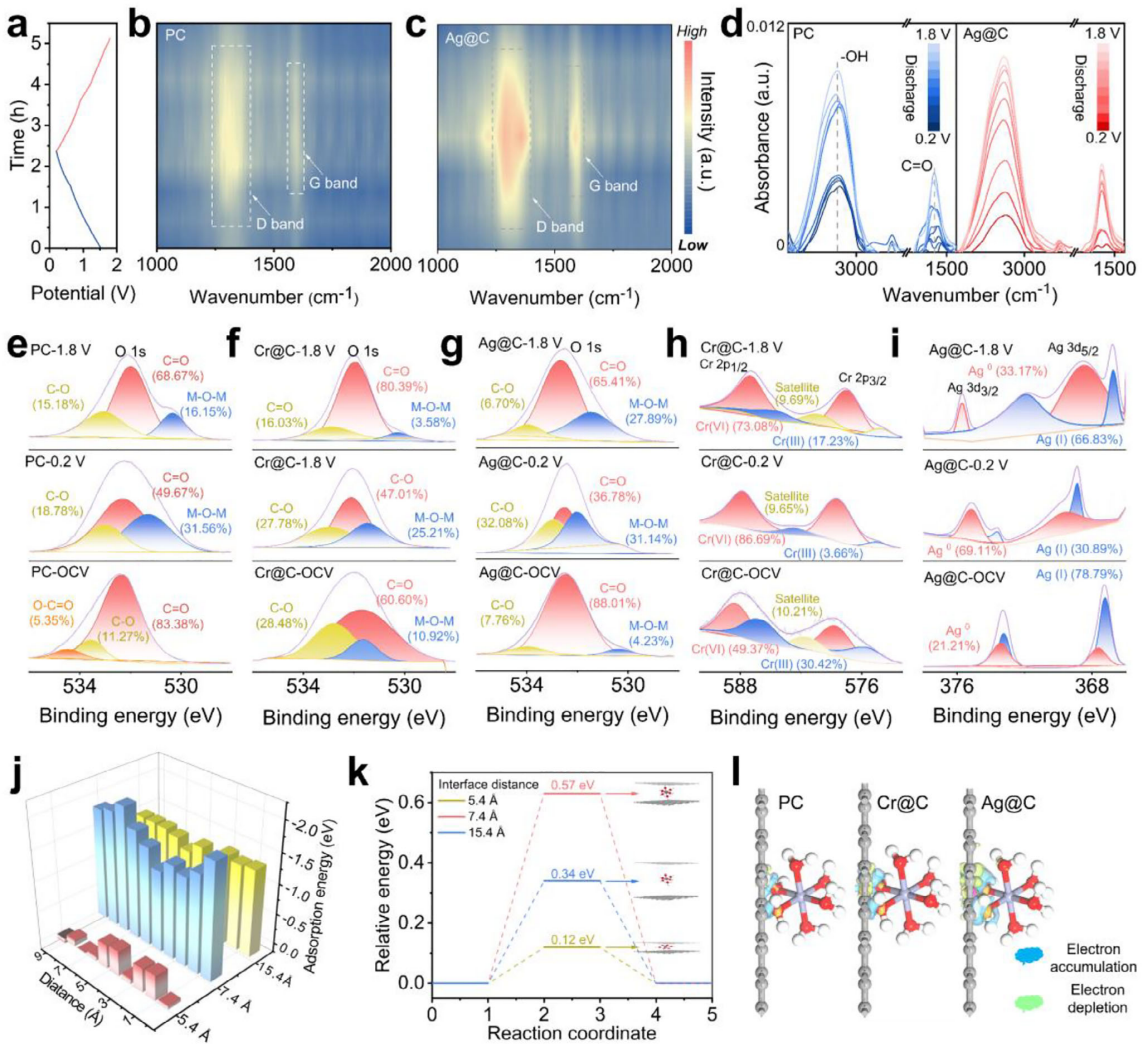
The discharge and charge curves of Ag@C at 0.2 A/g (a), in situ Raman spectra of PC and Ag@C electrode during charging–discharging process (b, c), ex situ FT‐IR spectra of PC and Ag@C electrode during charging–discharging process (d), high‐resolution O element spectrum of PC, Cr@C and Ag@C electrode collected in the stage of charge and discharge (e–g), high‐resolution Cr element spectrum of Cr@C electrode collected in the stage of charge and discharge (h), high‐resolution Ag element spectrum of Ag@C electrode collected in the stage of charge and discharge (i), adsorption energy and relative energy of [Zn (H_2_O)_6_]^2+^ in graphite layer with different interface distance (j,k), electron accumulation and depletion of PC, Cr@C and Ag@C electrode (l).

Ex situ Raman and XPS analyses of carbon anodes (PC, Cr@C, Ag@C) were performed to decode the charge storage mechanism. The evolution of D peak (1350 cm^−1^) and G peak (1580 cm^−1^) revealed distinct structural changes within the micropore upon the transition from the discharge state (0.2 V) to the charge state (1.8 V) (Figure 4a,b) [28]. For the starting PC anode, the slightly increased D peak intensity during the discharge process suggested the limited Zn‐ion diffusion within narrow micropore channels [23]. The full recovery of D peak intensity at 1.8 V, indicated reversible charge storage behavior. A similar tendency of D peak intensity was observed for the Cr@C anode (Figure S24), implying that Zn‐ion diffusion was not significantly enhanced. In contrast, the remarked increase of D peak intensity for Ag@C remarkably increased during the discharge stage (Figure 4c) corroborated the intensive ultra‐micropore filling by naked Zn‐ions. This observation was consistent with the enhanced EDLC distribution rate and improved capacitance retention rate under high current density conditions of Ag@C anode. Furthermore, G peak intensity increased for both Cr@C and Ag@C anodes due to the strengthened electron transfer within the graphene layer.

The ex situ FT‐IR spectrum of the Ag@C electrode was conducted to investigate the form variation of solvated Zn‐ion during the discharge and charge process (Figure 4d). The ─OH stretching vibration (3000–3500 cm^−1^) in the ex situ FT‐IR spectrum could be attributed to the coordinated H_2_O of the hydrated Zn ion [13]. The periodic decrease in ─OH peak intensity on PC spectra indicated the one‐step de‐solvation of the hydrated Zn ion. In contrast, the gradual intensity reduction during discharge for the Ag@C electrode suggested the stepwise de‐solvation of hydrated Zn ion, ultimately leading to naked Zn ion entering the ultra‐micropore channel. Concurrently, the attenuated ─C = O (1550–1700 cm^−1^) peak reflected the coordination effect with the naked Zn ion [29]. This process favored the subsequent formation of C─O─Zn bonds on the Ag@C pore wall and the generation of EDLC.

To probe the stepwise de‐solvation of hydrated Zn‐ion during the charge/discharge process, ex situ XPS analysis was performed on the starting PC and modified carbon anodes at different states (Figure S25). Specially, characteristic peaks for S and Zn elements emerged in the XPS spectrum of the total survey. Furthermore, Ag@C anode exhibited the highest relative content of Zn element at the discharge state, which was consistent with its strengthened Zn‐ion storage capability (Figure S26). Analysis of the C 1s spectra revealed a reduced relative content of C = O group and the appearance of C─O─M group at discharge stages. This transformation could be attributed to the combination between de‐solvated Zn‐ion and OFGs (Figure S27) [30]. A similar tendency was observed on the O 1s spectra of PC and modified carbon electrode at discharge/charge stages (Figure 4e–g). Notably, Ag@C at 0.2 V delivered the highest relative content of C─O─M (40.96%), which suggested the augment Zn‐ion coordination and enhanced pseudocapacitance. The form transformation of Cr/Ag element on the spectra of Cr 2p and Ag 3d indicated an electron support effect that facilitated the Zn‐ions stripping/deposition behavior (Figure 4h,i) [11].

DFT calculations elucidated the critical role of micropore channels dimension in modulating [Zn (H_2_O)_6_]^2+^ diffusion kinetic. For a micropore diameter of 0.56 nm, the [Zn (H_2_O)_6_]^2+^ adsorption energy was calculated as −0.41 eV (Figure 4j and Figure S24). In contrast, the micropore channel with diameters of 0.82 and 1.54 nm displayed enhanced adsorption energy and binding energy (Figure 4k). These results aligned with experimental rate performance, suggesting the fact that expanded micropore dimension facilitated [Zn (H_2_O)_6_]^2+^ diffusion. The analysis of the Differential Charge Density showed distinct electronic restructuring during discharge/charge. In the starting PC, the sparse electron accumulation at [Zn (H_2_O)_6_]^2+^ adsorption sites correlated with low pseudocapacitive contributions (Figure 4l). By contrast, the Ag (I)/Ag^0^ loading induced charge redistribution and formed C─O─Zn bonds with lower formation energy. This enhanced interfacial electric field could accelerate [Zn (H_2_O)_6_]^2+^ diffusion kinetics, which was evidenced by pseudocapacitive peaks near adsorption sites (ΔG = −0.62 eV). The synergistic effects of micropore channel expansion and electronic activation were further validated by operando electrokinetic analysis. The micropore with 0.74 nm diameter exhibited a DFT‐predicted diffusion coefficient of 2.8 × 10^−12^ cm^2^/s for [Zn(H_2_O)_6_]^2+^, which was 2.3 times faster than that of the 0.54 nm channel (1.2 × 10^−12^ cm^2^/s) [30]. These dual enhancements in electron conduction and ion diffusion underpin Ag@C superior supercapacitor performance.

### 2.4 The Cycling Stability and Zn‐ion Stepwise De‐solvation Mechanism of Ag@C

**FIGURE 5:**
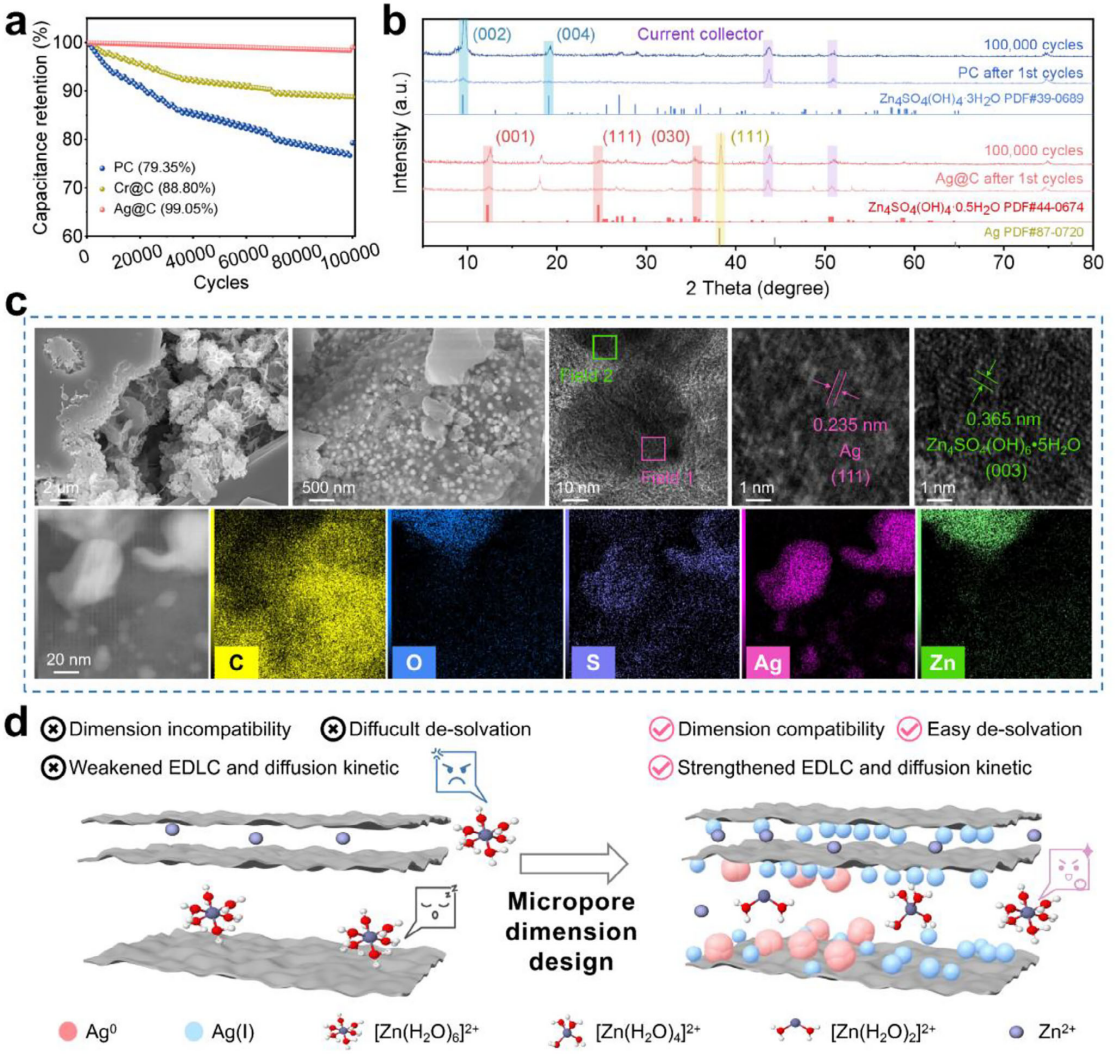
The cycling performance of PC and modified carbon electrodes (a), XRD patterns of PC and Ag@C electrode after 1st and 100000 times cycle (b), SEM, TEM, HR‐TEM and corresponding element mapping of Ag@C electrode after collected at 0.2 V discharge state after100000 cycles (c), and schematic diagram of Zn‐ion stepwise de‐solvation in the tightened micropore (d).

Cycling stability of ZiHSC was evaluated through galvanostatic charge–discharge tests (100000 cycles at 10 A/g). The starting PC exhibited a significant capacity decay of 79.4% retention rate (Figure 5a), which was attributed to incomplete Zn^2+^ adsorption/desorption in its narrow micropores. The moderate retention of 88.8% on Cr@C electrode may be due to the redox instability of Cr (VI)/Cr (III) compounds under prolonged cycling. Ag@C anode delivered the excellent capacity stability with 99.1% retention, which suggested that the fact that stepwise de‐solvation behavior of hydrated Zn‐ion was reversible and facilitated for adsorption sites activity.

In order to investigate the structure variation after the cycling process, the XRD pattern of Ag@C and PC within electrodes was conducted at the complete discharge state after 1st and 100000 times cycle (Figure 5b). In detail, the characteristic peak near 9.5° on PC patterns was assigned to Zn_4_SO_4_(OH)_4_•3H_2_O crystalline with (002) plane after 1st cycle, and intensified with the prolongation of work time [24]. In contrast, characteristic peaks of Zn_4_SO_4_(OH)_4_•0.5H_2_O crystalline at around 12.4° appeared on the Ag@C patterns after 1st and 100000 times cycle. This decreased hydrate number (between 0 and 1) of Zinc hydroxide sulfate (ZHS) hydrate crystalline in Ag@C anode demonstrated that the stepwise de‐solvation promoted the generation of naked and partially de‐solvated Zn ion. In addition, the characteristic peak near 38.2° on the Ag@C pattern was attributed to (111) plane of Ag NP, and its high intensity after 100000 cycles indicated the stable conductive network on porous carbon matrix [16].

XPS analysis was performed to examine the composition of the Ag@C electrode at 0.2 V discharge state after 100000 times cycle. Compared with the fresh Ag@C sample, the distinct Zn 2p characteristic peaks were observed on the total XPS spectrum with a relative element content of 16.9% (Figures S22 and S28a). The relative content of C─O in both the C 1s spectrum (16.2%) and O 1s spectrum (77.5%) significantly increased (Figure S28b,c). Meanwhile, the 23.1 eV spin energy separation of Zn 2p characteristic peaks on the Ag@C electrode was consistent with that of Zn─O [15]. This variation suggested the capacitance formation from the interaction between Zn ion and OFGs with the pore channel in the Ag@C anode. Furthermore, the coexistence of Ag NP and Ag^+^ on the Ag 3d spectrum demonstrated the stability of the conductive network (Figure S28e) [19].

The morphology of Ag@C electrode after 100000 times cycle was further observed by conducting SEM (Figure 5d). The images showed the deposition of the ZHS hydrate crystalline sheet at the discharge state, which was consistent with the XRD results. Meanwhile, spherical Ag NPs were uniformly distributed without noticeable aggregation (Figure S2). Furthermore, HR‐TEM images displayed clear lattice fringes corresponding to both Ag NPs and ZHS. The observed lattice spacing of 0.235 nm, assigned to the (111) plane of Ag NPs confirmed the excellent structure stability of conductive networks (Figure S29). In addition, the lattice fringe with an interplanar spacing of 0.364 nm matched the (003) plane of Zn_4_SO_4_(OH)_6_•0.5H_2_O [9]. Corresponding element mapping suggested that the well‐dispersed Ag NPs served as highly effective conductive agents, enhancing the diffusion kinetic of Zn ion (Figure 5e).

## 3 Conclusion

The modulation strategy of micropore dimension was proposed to synergistically address the dual challenges of low‐rate capability and sluggish reaction kinetics of Zn‐ion storage in porous carbon anode. By expanding the micropore diameter of PC and inducing stepwise de‐solvation behavior of hydrated Zn‐ions, this approach reduced the de‐solvation energy barrier of hydrated Zn‐ion and enhanced EDLC behavior. This advantage further enhanced Zn‐ion diffusion kinetic and capacitance contribution rate. In situ Raman, ex situ FT‐IR, and ex situ XPS revealed the strengthened Zn‐ion storage and electron supply in the expanded micropore channel during the discharge and charge process. The modified carbon anode exhibited a specific capacity of 224.1 mAh/g, achieved an impressive energy density of 179.6 Wh/kg (based on active material), and maintained 99.1% capacity retention after 100000 cycles in ZiHSC system. This approach established a novel paradigm for designing high‐performance carbon anodes and offered significant insights for optimizing Zn‐ion dynamics to advance energy storage systems.

## Author Contributions

Q.Q. performed conceptualization, visualization, and writing – original draft; Z.‐Z.C. performed conceptualization and formal analysis; Z.‐W.W. performed resources, data curation, and software; J.Q.X. performed data curation and validation; L.Q. performed conceptualization and writing – review and editing; F.G. performed writing – review and editing; and M.‐Q.Z. performed conceptualization, supervision, project administration, funding acquisition, and writing – review and editing.

## Conflicts of Interest

The authors declare no conflicts of interest.

## Supporting information




**Supporting File**: advs73798‐sup‐0001‐SuppMat.docx.

## Data Availability

Data will be made available on request.
