# Supplementary material for: Stepwise De‐solvation and Diffusion Kinetics of Hydrated Zn‐ion in Hierarchical Porous Carbon Anode for Improved EDLC Behavior
Source: Adv Sci (Weinh). 2026 Feb 3;13(17):e19684. doi: 10.1002/advs.202519684 (PMC13042696; doi:10.1002/advs.202519684)
Supplement: Supplementary file 1 — Supporting File: advs73798‐sup‐0001‐SuppMat.docx. [file ADVS-13-e19684-s001.docx]

**Supporting information**

**Stepwise De-solvation and Diffusion Kinetics of Hydrated Zn-ion in Hierarchical Porous Carbon Cathode for Improved EDLC Behavior**

*Qiang Qu ^a^, Zhi-Zhen Chi ^b^, Zhi-Wen Wang ^b^, Jia-Qing Xie ^a^, Ling Qiu ^a^, Fang Gu ^a^, Ming-Qiang Zhu ^a, b, c, d *^*

*^a^ College of Mechanical and Electronic Engineering, Northwest Agriculture & Forestry University, Yangling 712100, China*

*^b^ College of Forestry, Northwest Agriculture & Forestry University, Yangling 712100, China*

*^c^ Key Laboratory of Eucommia of National Forestry and Grassland Administration, Yangling 712100, China*

*^d^ Key Laboratory of Shaanxi Province on Development and Utilization of Economic Plant Resources, Yangling 712100, China*

Corresponding E-mails: [zmqsx@nwsuaf.edu.cn](mailto:zmqsx@nwsuaf.edu.cn).

**Table of Contents**

List of Supplementary Experimental section……………………………… … page 3

List of Supplementary Tables………………………………………………… .page 10

List of Supplementary Figures…………………………………………………page 12

**List of Supplementary Manuscript**

*Materials and agents: Eucommia ulmoides* Wood (EUW) and derived porous carbon (PC) were prepared following the previous work [1]. In detail, the wood feedstock was collected from Northwest Agriculture and Forestry University of Yangling district in Shaanxi province, China, and PC material was prepared by phosphoric acid activation method. All chemicals (K_2_Cr_2_O_7_, AgNO_3_, Zn_2_SO_4_) in analytical grade (99.999%) were purchased from Aladdin Biochemical Pharmaceutical Co., Ltd (Shanghai, China).

*Porous carbon material modification experiment:* Cr (VI) and Ag (I) solutions were prepared by dissolving above-mentioned agents in water with a concentration of 20 mg/L [2]. In order to examine the adsorption property, 40 mg PC adsorbent was added into 150 mL conical flask containing 100 mL solution. The mixed slurry was then stirred under the speed of 120 rpm and temperature of 308 K in the dark environment. The supernatant was sampled to measure the residual concentration of pollutants, and the residual solid was collected, froze, and dried for 48 h, which were then successively named as Cr@C and Ag@C. Besides, the adsorption isotherm was conducted at different initial concentrations of 10 - 250 mg/L at 308 K. After 24 h, the mixture was transferred to centrifuge tube and centrifuged with 10,000 rpm for 10 min.

*Adsorption quantity analysis*: In the above experiments, the concentration of residual solution was detected by Graphite Furnace Atomic Absorption Spectrometer (PinAAcle 900 T, PerkinElmer, America) and UV-spectrophotometer. The adsorption capacities of PC adsorbent for the various pollutants were calculated using Eq. (S1), (S2). The adsorption isotherm data was simulated by the Langmuir (Eq. (S3)) and Freundlich (Eq. (S4)) isotherm model, respectively. The characteristics of the adsorption process between the adsorbent and pollutants ions can be expressed in terms of a dimensionless constant or separation factor (*R*_L_) by Eq. (S5):

$\text{Q}_{\text{e}}\text{=}\frac{\left( \text{C}_{\text{0}}\text{-}\text{C}_{\text{e}} \right)\text{V}}{\text{m}}$ (S1)

$R_{e}\text{=}\frac{\left( \text{C}_{\text{0}}\text{-}\text{C}_{\text{e}} \right)}{\text{C}_{\text{0}}}\text{×100\%}$ (S2)

$\text{Q}_{\text{e}}\text{=}\frac{\text{Q}_{\text{max}}C_{0}\text{K}_{\text{L}}}{\text{1+ }\text{C}_{\text{0}}\text{K}_{\text{L}}}$ (S3)

$\text{Q}_{\text{e}}\text{= }\text{K}_{\text{F}}\text{C}_{\text{0}}^{\text{1/n}}\text{ }$ (S4)

$\text{R}_{\text{L}}\text{=}\frac{\text{1}}{\text{1+}\text{K}_{\text{L}}\text{C}_{\text{0}}}$ (S5)

where *C_0_*, and *C_e_* were Cr (VI)/ Ag (I) concentration (mg/L) at the beginning time, and equilibrium time, respectively, *V* was the volume of the solution (L), *m* was the dry weight of the measured adsorbents (g). *Q_max_* was the maximum removal capacity (mg/g), *K_L_* was an equilibrium constant (L/mg), *R_L_* was a dimensionless constant or separation factor, *n* represented the bond distribution (dimensionless), which was the heterogeneity factor, and *K_F_* was the Freundlich constant that related to the removal ability (mL^3^/g). All adsorption experiments were conducted in duplicate with well reproducible results. The reported data was obtained by averaging the values of two replicated experiments and error bars represent the standard deviation of the average.

*Measurements of supercapacitive performance:* PC and modified carbon products served as the active material in the electrode, and the supercapacitive properties were evaluated in the 2032 button cell. The composite films containing an active material loading of ~2 mg/cm^2^ were coated on copper foil as cathode electrode, and the zinc foil was employed as the anode electrode. Meanwhile, 1 M ZnSO_4_ solution and glass fiber was employed as the electrolyte and separator.

*Supercapacitive performance analysis:* All the CV experiments were conducted on an electrochemical working station (CHI660E, Shanghai, China). The potential window was ranged from 0.2 to 1.8 V. EIS technique was performed on an electrochemical work station (CHI660E, Shanghai, China) and the frequency was ranged from 100 kHz to 10 mHz with an ac signal amplitude of 5 mV at open circuit potential. GCD experiments were carried out by a Land Battery test System (Land, PR China) and the charging-discharging current densities were ranged from 0.2 to 5 A/g. The calculation methods of specific capacitance (*Cs*, F/g), energy density (*E*, Wh/kg) and power density (*P*, W/kg) were shown as following,

$\text{C}_{\text{s}}\text{=}\text{I}\text{×}\text{∆}\text{t}\text{/(}\text{m}\text{×}\text{∆}\text{v}\text{)}$ (S6)

$\text{C}_{\text{s}}\text{=}\text{Q}\text{/(}\text{m}\text{×∆}\text{v}\text{)}$ (S7)

$\text{E}\text{=0.5}\text{C}_{\text{s}}\text{Δ}\text{V}^{\text{2}}\text{/3.6}$ (S8)

$\text{P}\text{=3600}\text{E}\text{/}\text{Δ}\text{t}$ (S9)

where *I* was the current (A), m was the weight of activated material (g), *Δt* was the charge and discharge time, *Q* was fitting area of CV curves, and Δ*V* was the voltage window. The CV curve of the electrodes was obtained at different scanning rates (5, 10, 20, 50 mV/s). The EIS data were collected at a frequency between 100 kHz and 10 mHz with an AC signal amplitude of 5 mV.

Zn^2+^ diffusion coefficient (cm^2^ s^−1^) was calculated via GITT curves according to the following equations:

$\text{D}\text{=}\frac{\text{R}^{\text{2}}\text{T}^{\text{2}}}{\text{2}\text{An}^{\text{4}}\text{F}^{\text{4}}\text{C}^{\text{2}}\text{σ}^{\text{2}}}$ (S10)

where R is gas constant; *T* is kelvin temperature; *A* is electrode area; *n* is number of electrons transferred during the reaction; F is Faraday constant; *σ* is the slope of straight-line.

*Characterization of PC and modified carbon samples:* The morphology and structure of carbon materials were observed by Scanning Electron Microscopy, energy dispersive X-ray Detector (SEM-EDX) (TM3000, Hitachi, Japan), and Transmission electron microscope (TEM) (Talos F200S, Themo fisher, America). Specific surface area and aperture distribution tester (Tristar II 3020, Micromeritics, America) was used to quantify the pore parameters using the multipoint N_2_ adsorption-desorption technique at 77 K, and the results were computed using the Brunauer-Emmett-Teller (BET) model. The samples were degassed overnight (at 293K) prior to analysis [3]. The porosity was measured by Small Angle X-ray Scattering (SAXS) (Nanostar SAXS, Bruker, Germany), and the raw data was fitted by SASfit software (Version 0.94.11). The surface chemical properties were detected by using FT-IR (Vertex70, Bruker, Germany) between the wavenumber range from 4000 to 400 cm^-1^ at a resolution of 4 cm^-1^ [4]. The crystal structure was performed by X-ray powder diffraction (XRD) (D8 ADVANCE A25, Bruker, Germany) with a Cu-Kα radiation at 30 kV and 20 mA. The XRD patterns were examine by using JADE software (Version 5.0) [5]. The Raman spectra of samples were recorded at ambient temperature on a Raman microscope (Horiba JobinYvon, Longjumeau, France) equipped with a confocal microscope (Olympus BX51, Tokyo, Japan) and a motorized x and y stage with an argon-ion laser at an excitation wavelength of 532 nm [6]. Conductivity and Zeta potential were severally measured by the four probe low resistance/contact resistance tester (FT-541SJB-341, ROOKO, China) and the potential analyzer (Sur Pass 3, Anton Pa, Austria) [7]. The hydrophilia property was tested by WCA (OCA20, Dataphysics, Germany) [8].

*Theoretical Calculation*: First-principles calculation of the adsorption energy of hydrated zinc ions is as follows, all calculations were performed using Materials Studio (ver. 2020). The pore-size of porous carbon material was explicitly defined based on the interspace (5.4 Å, 7.4 Å, and 15.4 Å) within the bi-layer graphene model [9]. DFT calculations were performed using the DNP (Double Numerical plus Polarization) basis set [10]​. The electron exchange-correlation interactions were simulated by the projector augmented wave (PAW) pseudopotentials [11], with a plane-wave cutoff of 520 eV and the Perdew-Burke-Ernzerhof functional [12] within the Generalized Gradient Approximation (GGA-PBE) [5]. The convergence criteria for energy and force were set to 1×10^-5^ eV and 0.02 eV Å^-1^, respectively [13]. To properly describe the van der Waals (vdWs) interaction, Grimme’s Mol^3^ empirical correlation was used [10]. And spin polarization was included for all calculation. These parameters were chosen to balance accuracy and computational efficiency while maintaining high reproducibility standards. By constructing a graphene supercell containing 6×6-unit cells with a vacuum region thickness of 20 Å, the interaction between the plates was negligible. The adsorption energy is calculated based on the following formula (S11),

ΔE = E_A−B_ − E_B_ – E_A_ (S11)

where E_A-B_, E_B_, and E_A_ represent the energies of adsorbed H and no adsorbed H of defective structures containing pollutant atoms, and the energies of individual H, respectively.

The interaction energy (E_in_) of [Zn(H_2_O)_6_]^2+^ in double-layer graphene was calculated using the following equation for interlayer distances of 0.54 nm, 0.74 nm and 1.54 nm,

E_in_ = E(total) – E(graphene) - E[Zn(H_2_O)_6_]^2+^ (S12)

where E(total) represents the total energy of [Zn(H_2_O)_6_]^2+^ adsorbed on double-layer graphene, E(graphene), and E[Zn(H_2_O)_6_]^2+^ represent the energy of double-layer graphene, and [Zn(H_2_O)_6_]^2+^ ion, respectively.

The calculation of de-solvation energy is based on a cluster model under the B97-3c method. All geometric structures were fully optimized, and frequency calculations were performed to verify the structural validity and obtain corrected thermodynamic quantities. In the presence of Zn²⁺, the spin multiplicity of the model is always 2. All calculations were conducted using an implicit solvent model to simulate the aqueous environment. The successive de-solvation energy (E_d_):

E_d_=E{[Zn(H_2_O)_n−1_]^2+^} + E(H_2_O) - E{[Zn(H_2_O)_x_]^2+^} (x=1-6) (S13)

The adsorption energy (E_ads_) was calculated according to the following formula:

$\text{E}_{\text{ads}}\text{=}\text{E}_{\text{Zn/carbon}}\text{-}\text{E}_{\text{carbon}}\text{-}\text{E}_{\text{Zn}}$ (S14)

In this equation, E_Zn/carbon_, E_carbon_, E_Zn_ correspond to the energy of the carbon system with Zn adsorbed, the energy of the carbon system without Zn adsorption, and the energy of a single Zn atom in the bulk structure, respectively. The same procedure was applied to calculate the adsorption energy for SO_4_^2-^. A more negative value of adsorption energy indicates a stronger adsorption capacity of the ions on the carbon framework.

**List of Supplementary Tables**

**Table S1** The *Langmuir* and *Freundlich* adsorption isotherm models fitting parameters of PC and modified carbon samples.

| Pollutant | *Q*_e_ (mg/g) | *Langmuir* | | | | *Freundlich* | | |
| --- | --- | --- | --- | --- | --- | --- | --- | --- |
|  |  | *K*_L_  (L/mg) | *Q*_max_ (mg/g) | *R*_L_ | *R*^2^ | *K*_L_  (L/mg) | *Q*_max_ (mg/g) | *R*^2^ |
| Cr | 119.72 | 0.001 | 125.66 | 0.0910~  0.8511 | 0.9996 | 128.11 | 0.1848 | 0.9898 |
| Ag | 21.94 | 0.004 | 22.01 | 0.04873~  0.0987 | 0.9976 | 22.46 | 0.1115 | 0.9958 |

**Table S2** The element contribution in EDX-mapping on PC and modified carbon samples.

| Atomic Fraction (%) | PC | Cr@C | Ag@C |
| --- | --- | --- | --- |
| C | 92.6±1.38 | 88.56±1.84 | 92.93±1.46 |
| O | 7.4±1.38 | 8.05±1.86 | 6.06±1.45 |
| Cr | * | 3.39±0.21 | * |
| Ag | * | * | 1.01±0.01 |

* The value is less than 0.001%.

**Table S3** The fitting results of D peak and G peak in Raman spectrum of PC and modified carbon samples.

| Sample | D peak | | | G peak | | | I_D_/I_G_ |
| --- | --- | --- | --- | --- | --- | --- | --- |
|  | Position  (cm^-1^) | Intensity  (a.u.) | Area  (%) | Position  (cm^-1^) | Intensity  (a.u.) | Area  (%) |  |
| PC | 1364.78 | 4207.60 | 73.93 | 1587.81 | 4638.01 | 26.06 | 0.9071 |
| Cr@C | 1351.75 | 2556.32 | 71.98 | 1590.80 | 3495.18 | 28.01 | 0.7313 |
| Ag@C | 1348.58 | 2564.91 | 68.59 | 1591.14 | 3599.68 | 30.40 | 0.7248 |

**Table S4** The element contribution in XPS spectra survey of PC and modified carbon samples.

| Element contribution (%) | PC | Cr@C | Ag@C |
| --- | --- | --- | --- |
| C | 81.39 | 76.17 | 86.23 |
| O | 18.61 | 13.78 | 11.53 |
| Cr | * | 10.05 | * |
| Ag | * | * | 2.24 |

* The value is less than 0.001%.

**Table S5.** The fitting parameters of equivalent circuit models for PC, Cr@C and Ag@C EIS spectra.

| Samples | Rs ^c^ | Rct ^b^ | Wo ^a^ |
| --- | --- | --- | --- |
|  | (Ω) | (Ω) | (Ω) |
| PC | 0.58 | 40.49 | 15.69 |
| Cr@C | 0.55 | 15.67 | 9.57 |
| Ag@C | 0.25 | 3.21 | 2.34 |

^a^ Solution resistance was fitted by EIS spectrum using Z-View software.

^b^ Charge transfer resistance was fitted by EIS spectrum using Z-View software.

^c^ Warburg impedance was fitted by EIS spectrum using Z-View software.

**List of Supplementary Figures**


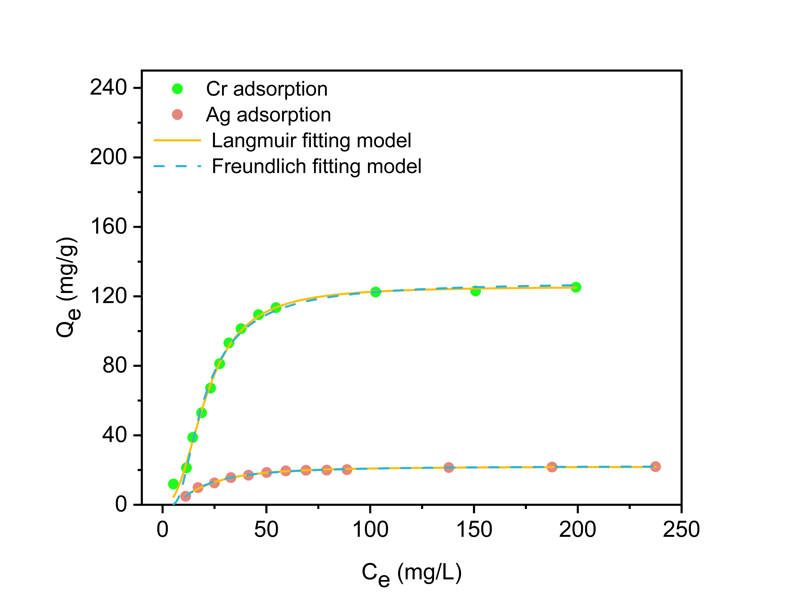


**Figure S1** The Langmuir and Freundlich adsorption isotherm curves of PC for different functional agents.


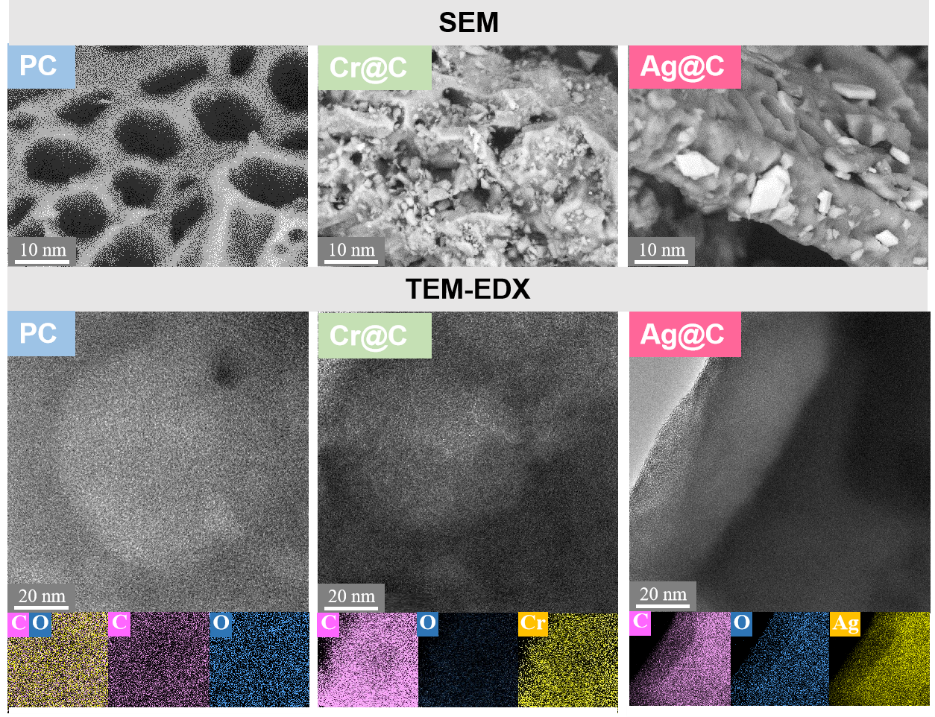


**Figure S2** The SEM images, TEM images and EDX-mapping of PC and modified carbon samples.


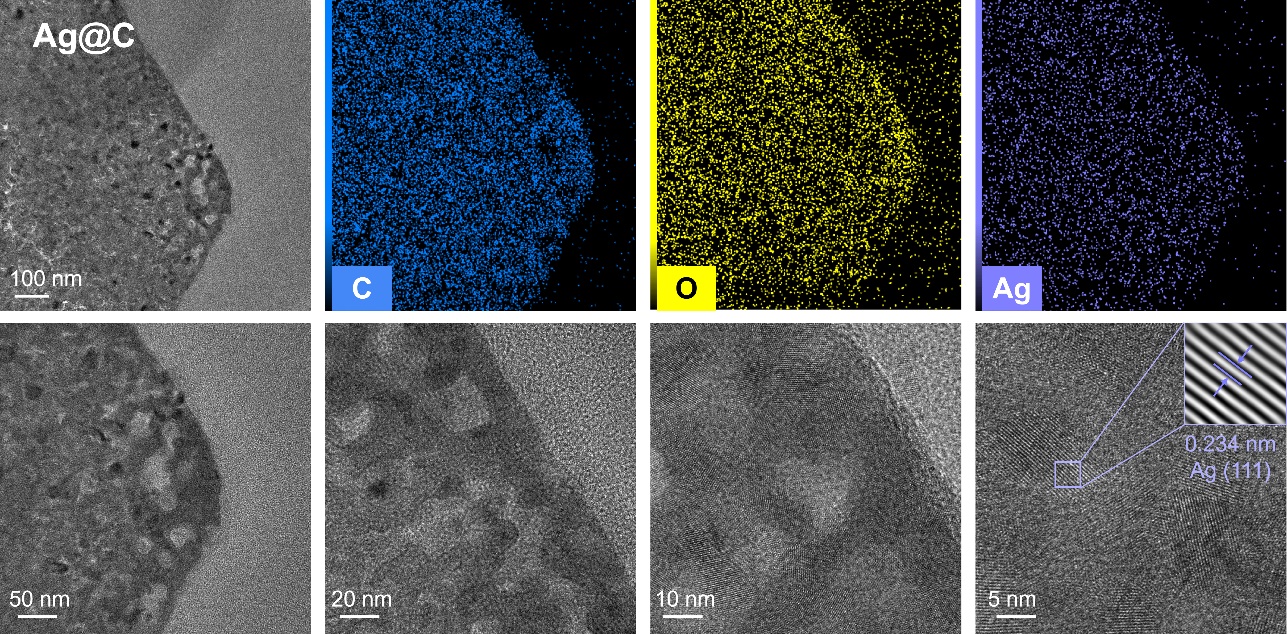


**Figure S3** The TEM and HR-TEM images of Ag@C with corresponding C, O, Ag element mapping images.


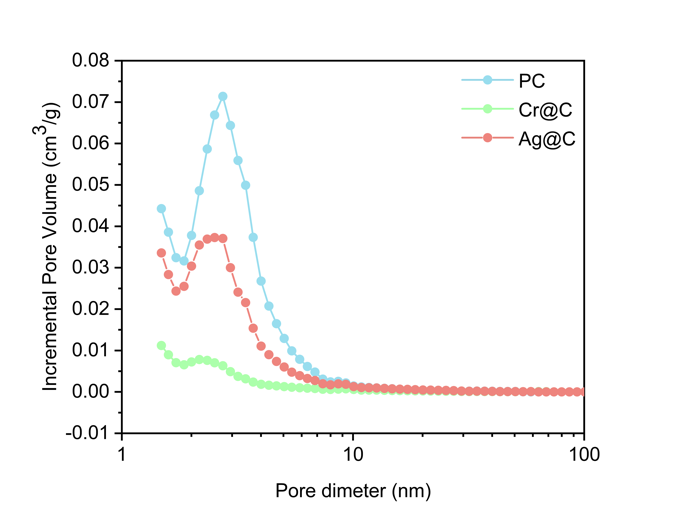


**Figure S4** The pore diameter distribution of PC and modified carbon samples.

**
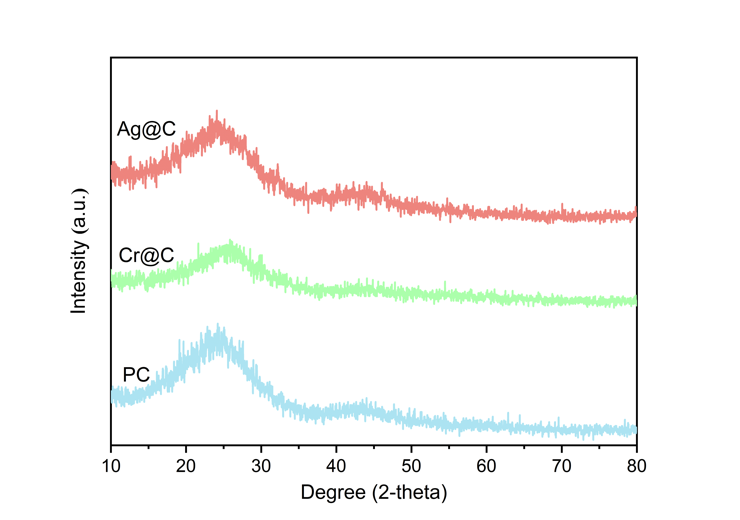
**

**Figure S5** The XRD spectrum of PC and modified carbon samples.


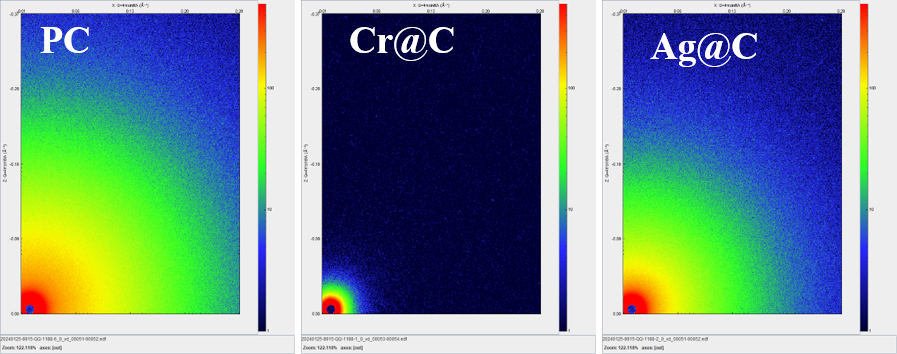


**Figure S6** The SAXS spectrum of PC and modified carbon samples.


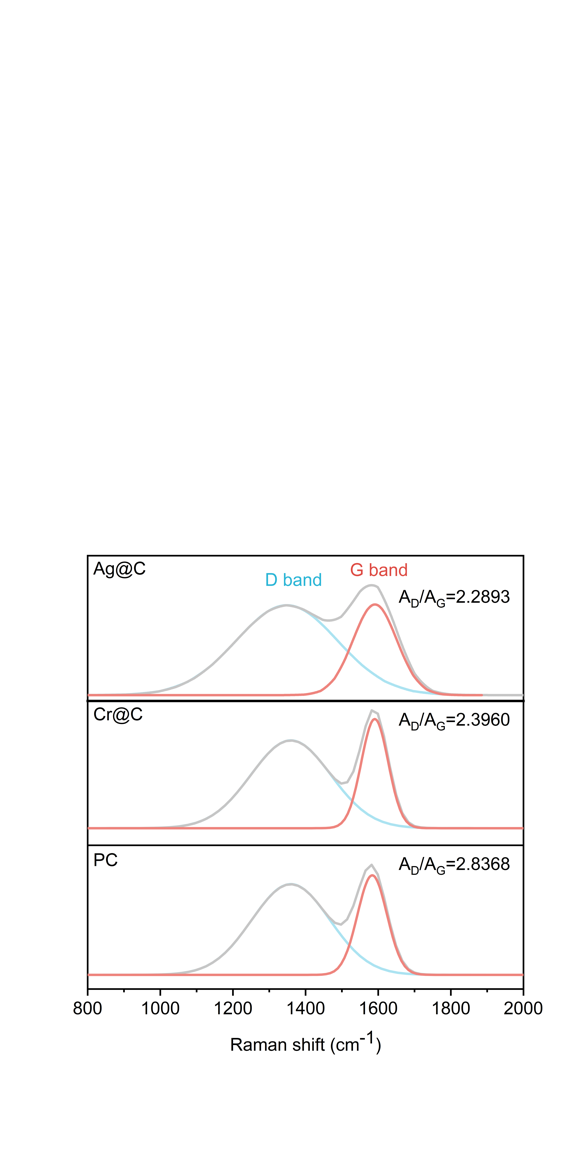


**Figure S7 The Raman spectrum of PC and modified carbon samples.**


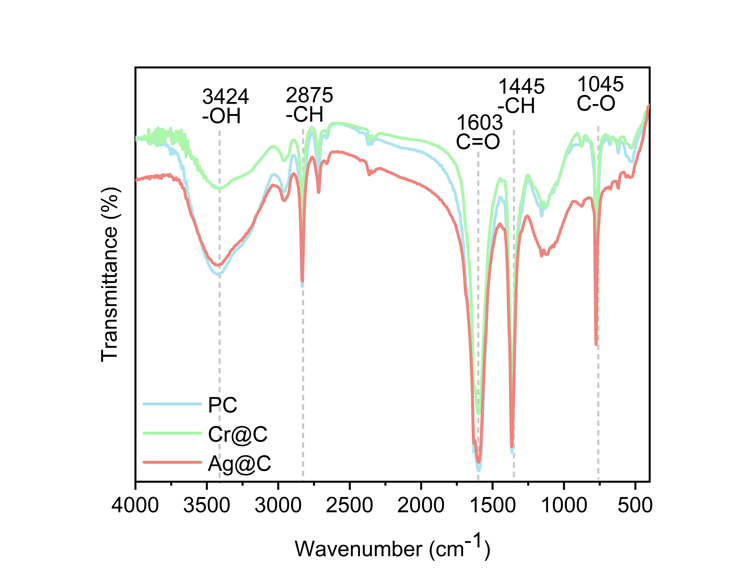


**Figure S8** The FTIR spectrum of PC and modified carbon samples.

**
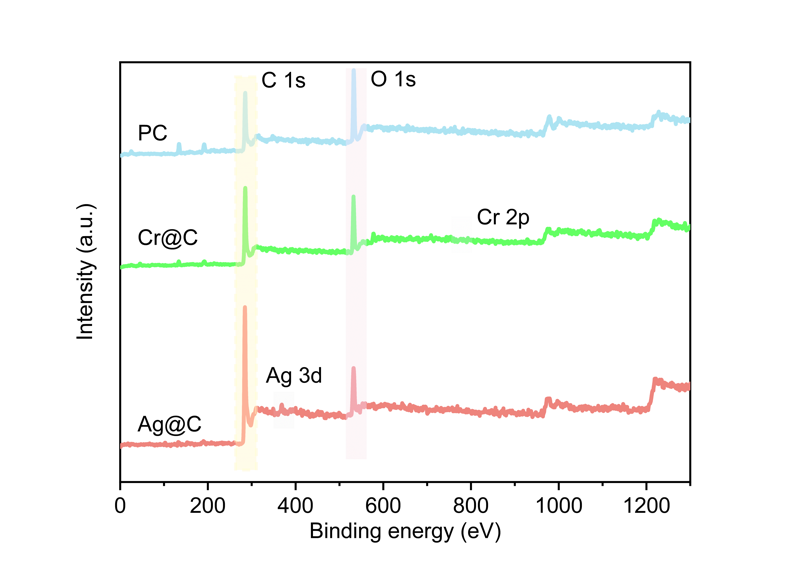
**

**Figure S9** The total XPS spectrum of PC and modified carbon samples.

**
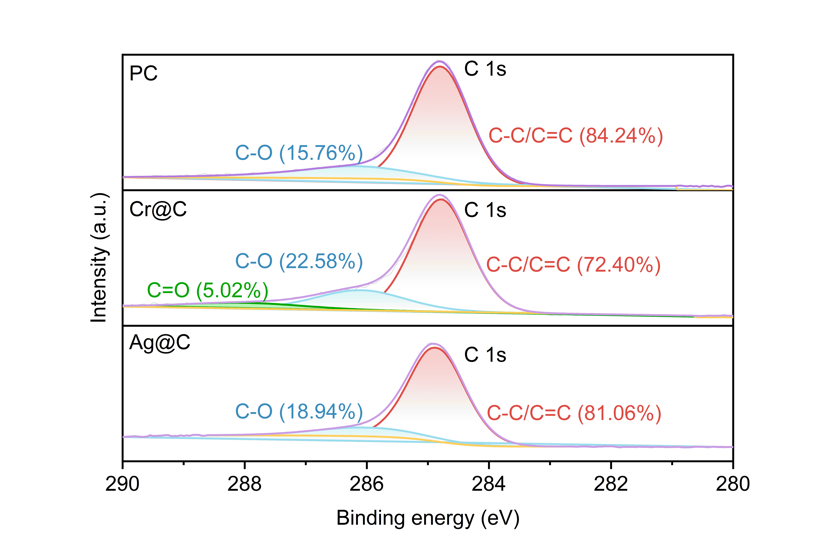
**

**Figure S10** The high-resolution C element spectrum of PC and modified carbon samples.

**
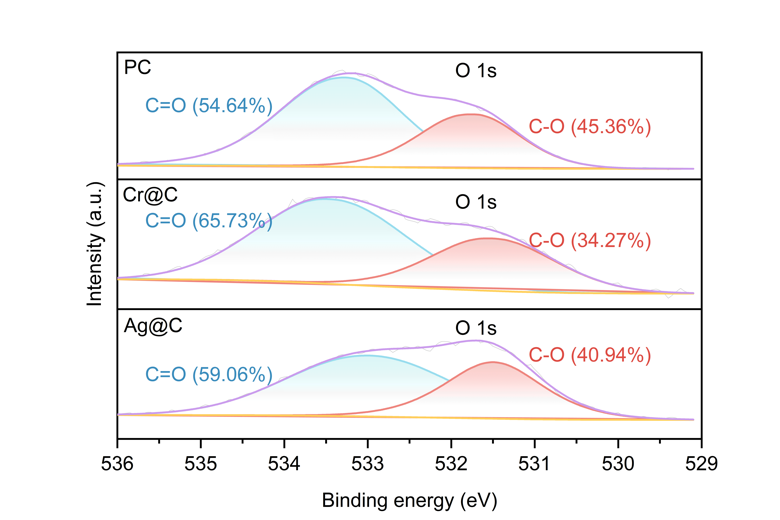
**

**Figure S11** The high-resolution O element spectrum of PC and modified carbon samples.


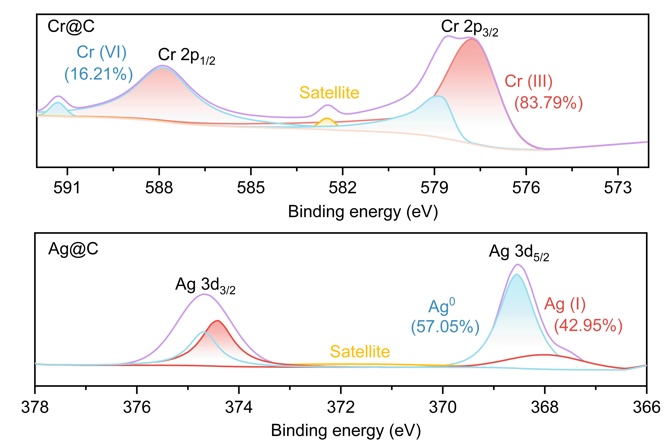


**Figure S12** The high-resolution Cr/Ag element spectrum of Cr@C/Ag@C.


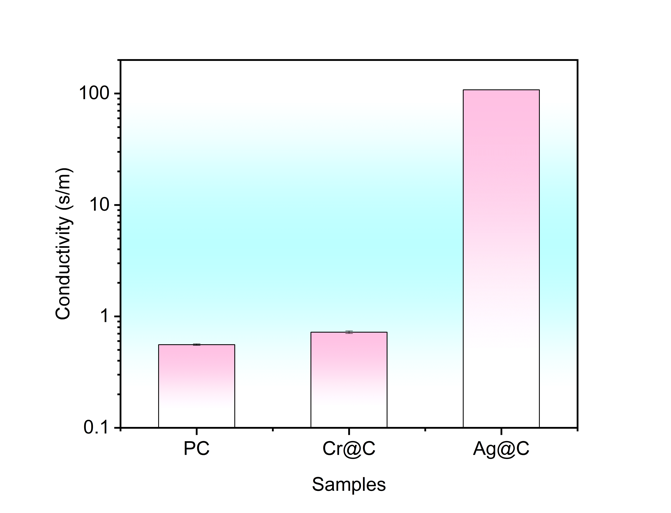


**Figure S13** The conductivity of PC and modified carbon samples


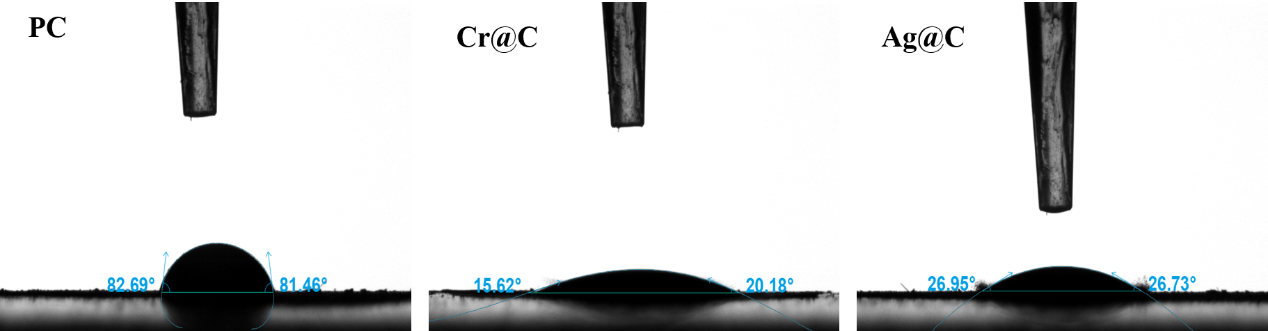


**Figure S14** The water contact angels of PC electrode and modified carbon electrodes.


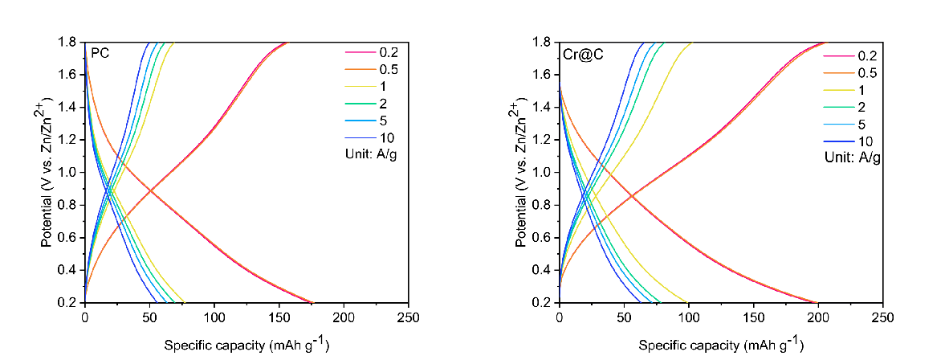


**Figure S15** The charging-discharging curves of PC electrode and Cr@C carbon electrodes with the current density ranging from 0.2 to 10 A/g in ZiHSC system.

**
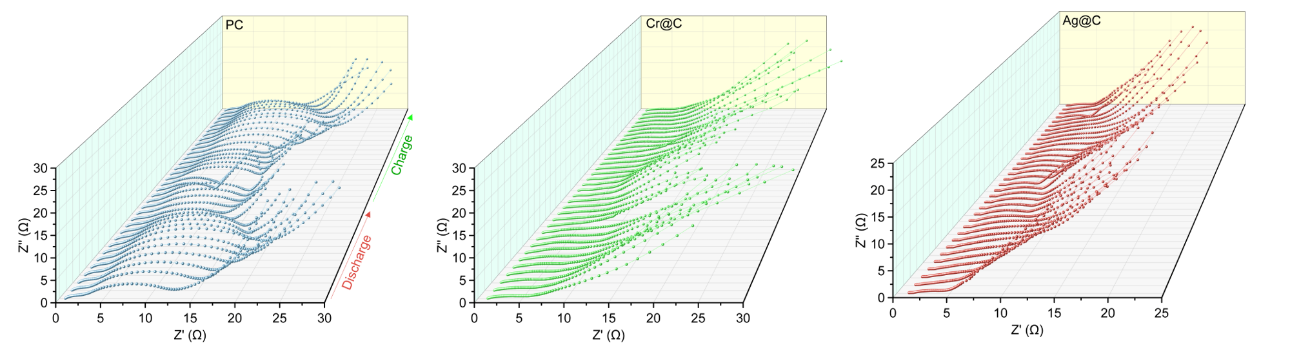
**

**Figure S16** The Nyquist plots of PC electrode and modified carbon electrodes during the discharge process in ZiHSC system.

**
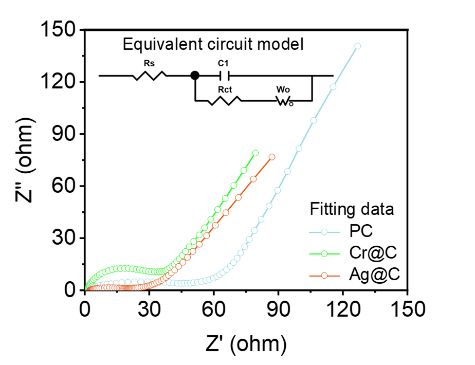
**

**Figure S17** The fitted EIS plots by equivalent circuit model.


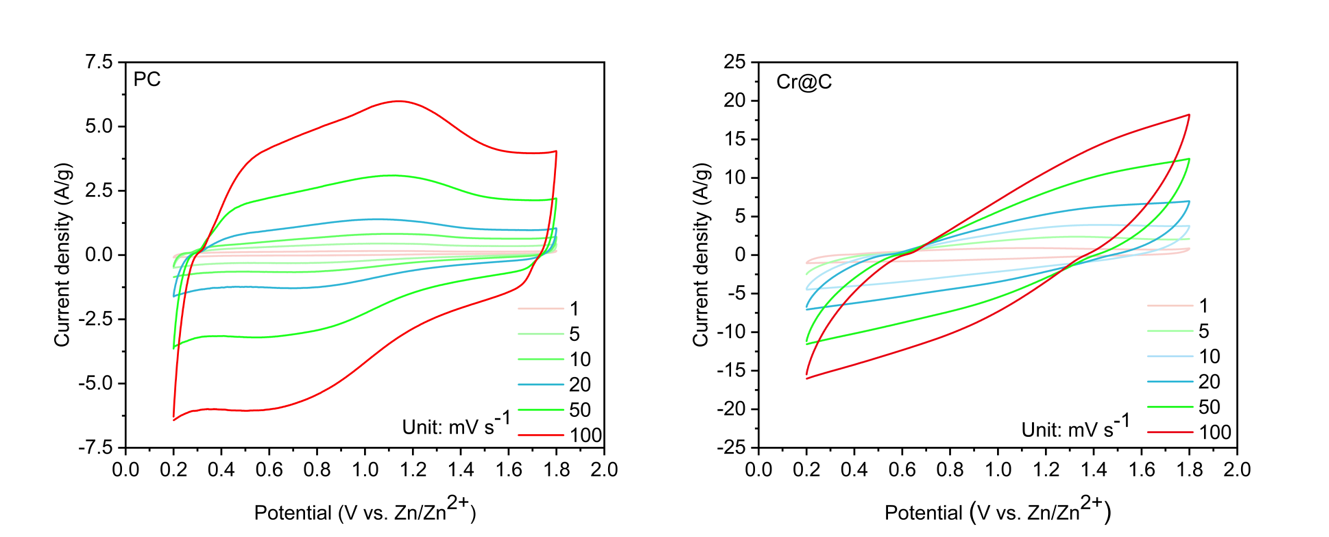


**Figure S18** The CV curves of PC electrode and Cr@C electrodes with the scan rate ranging from 1 to 100 mV/s in ZiHSC system.

**
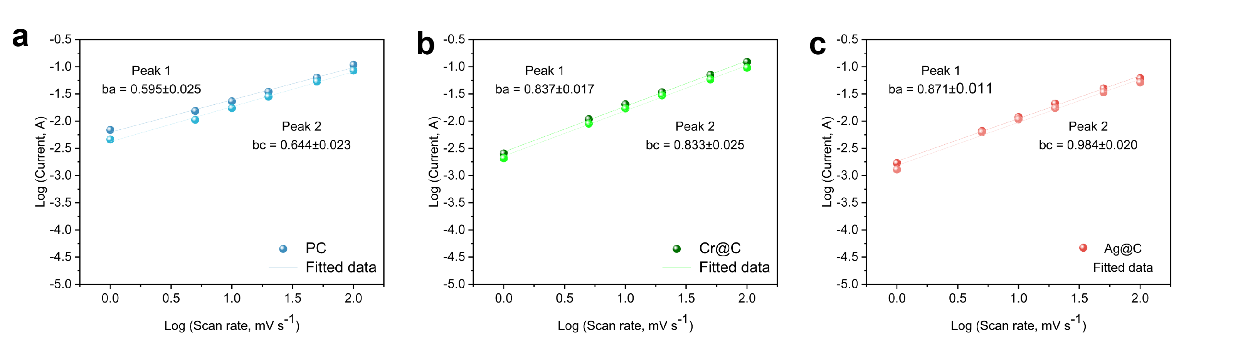
**

**Figure S19** The linear relationships between logarithm currents and logarithm sweep rate of PC electrode and modified carbon electrodes.


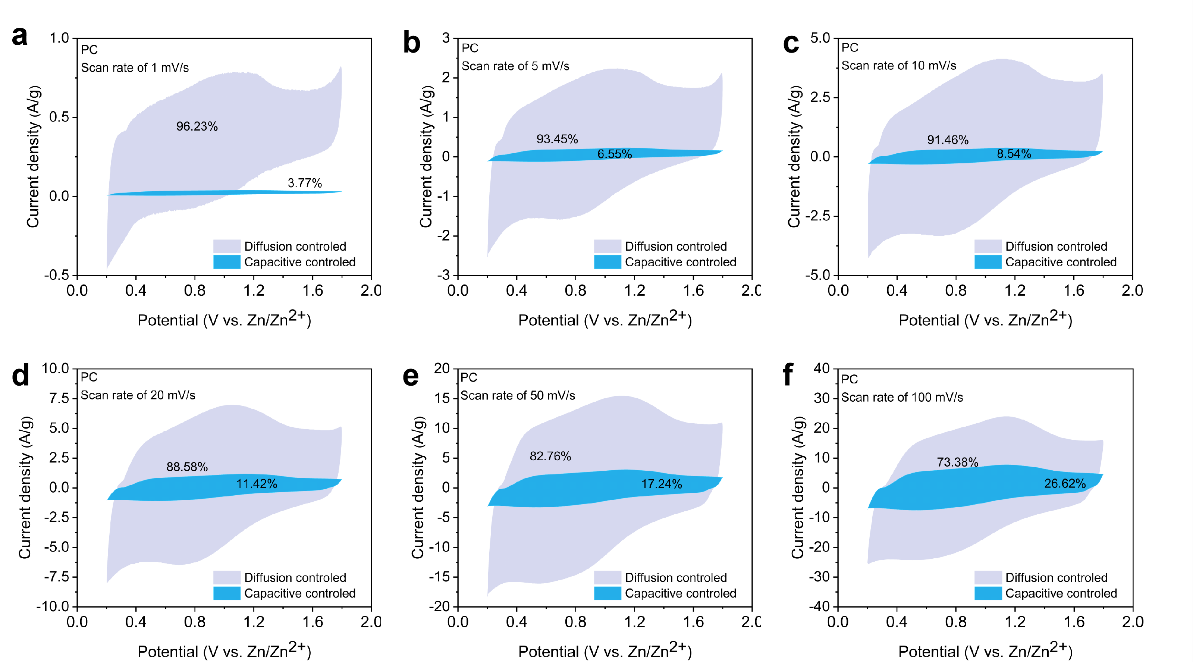


**Figure S20** The capacitance distribution of PC electrode with the scan rate ranging from 1 to 100 mV/s in ZiHSC system.


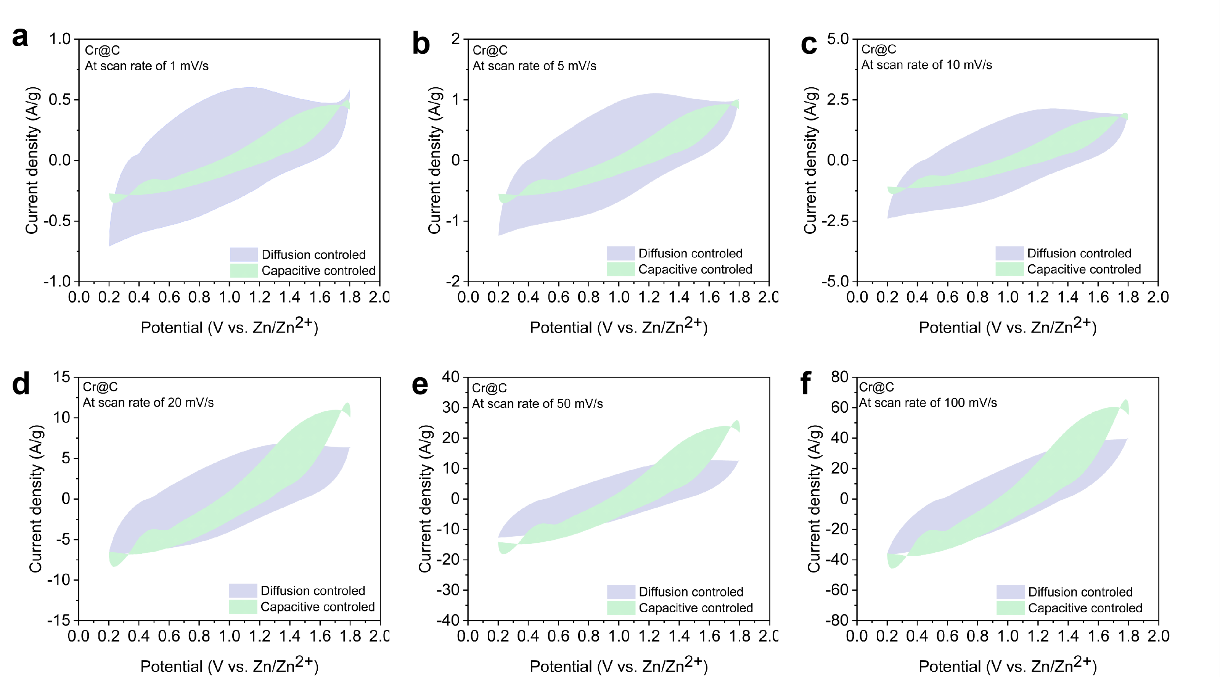


**Figure S21** The capacitance distribution of Cr@C electrode with the scan rate ranging from 1 to 100 mV/s in ZiHSC system.


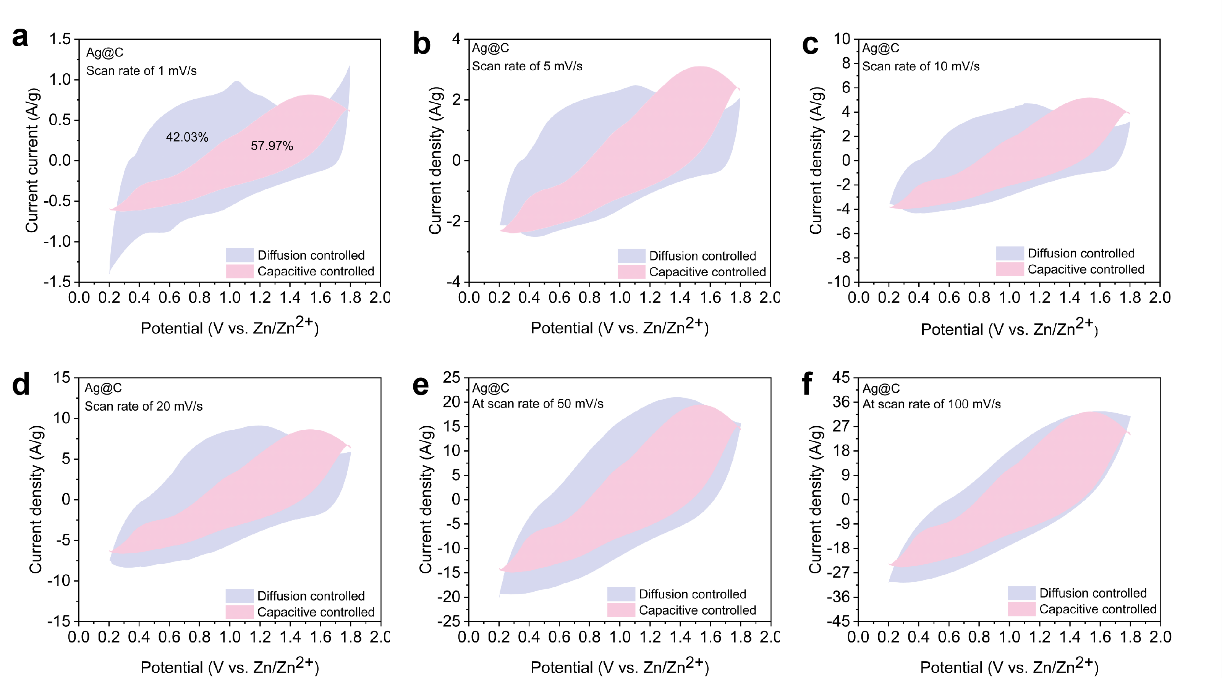


**Figure S22** The capacitance distribution of Ag@C electrode with the scan rate ranging from 1 to 100 mV/s in ZiHSC system.

**
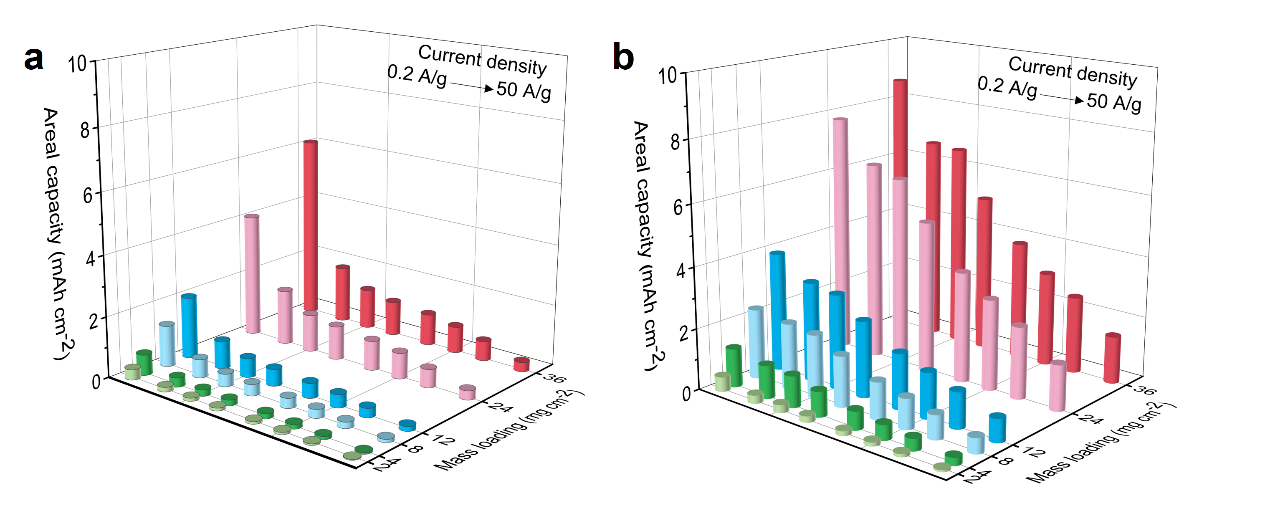
**

**Figure S23** The specific capacity at different current density of PC (a) and Ag@C electrode (b) with different mass loadings.

**

**

**Figure S24** The *in situ* Raman spectra of Cr@C electrode during discharge and charge process.

**
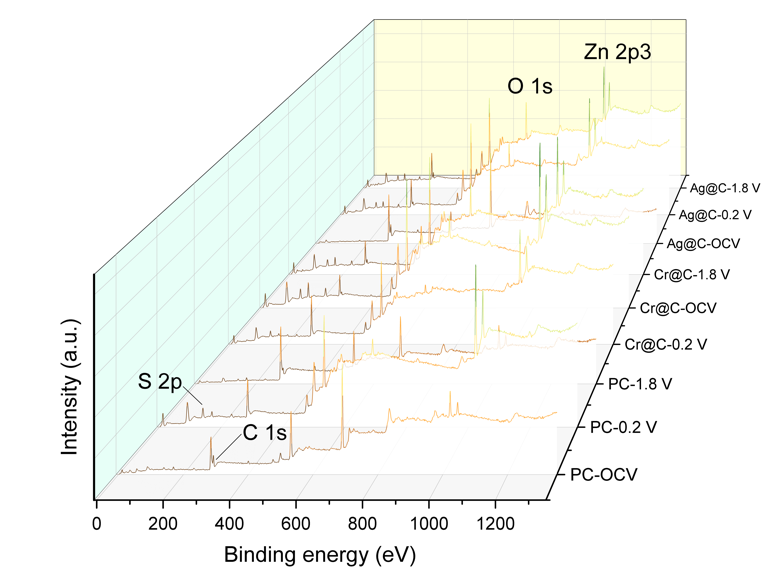
**

**Figure S25** The total XPS spectrum of PC electrode and modified carbon electrodes during the charging-discharging process in ZiHSC system.


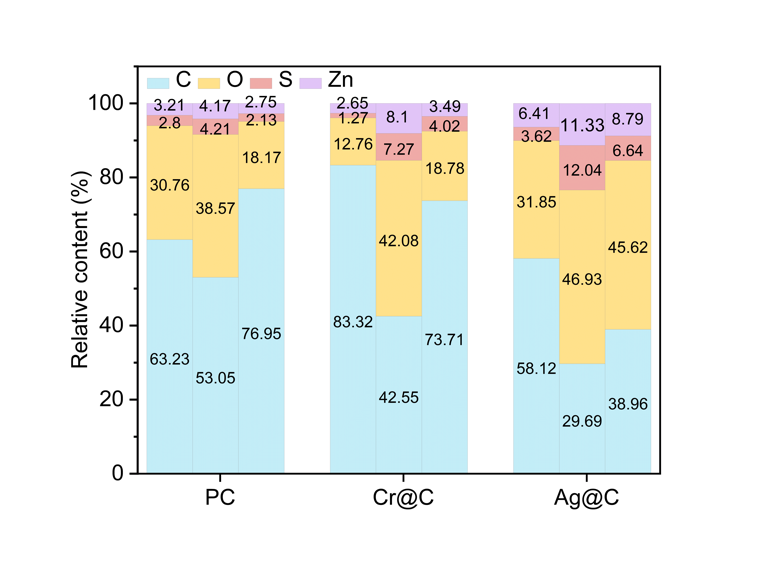


**Figure S26** The relative content of different elements on the surface of PC electrode and modified carbon electrodes during the charging-discharging process in ZiHSC system.


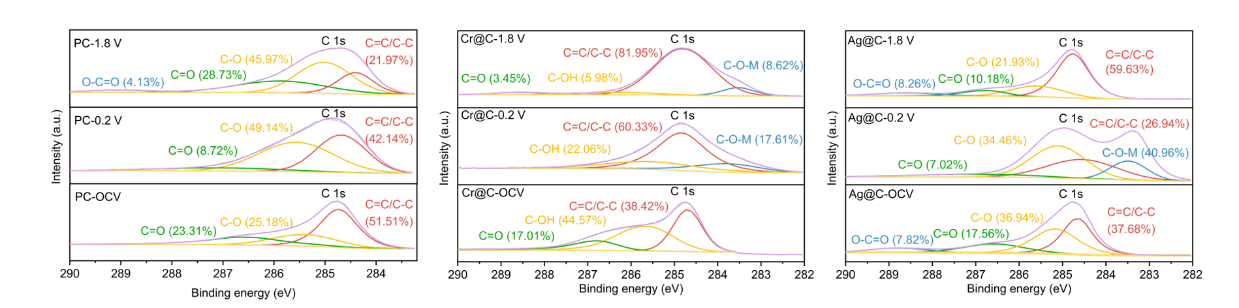


**Figure S27** The high-resolution spectrum of C element on the surface of PC electrode and modified carbon electrodes during the charging-discharging process in ZiHSC system.


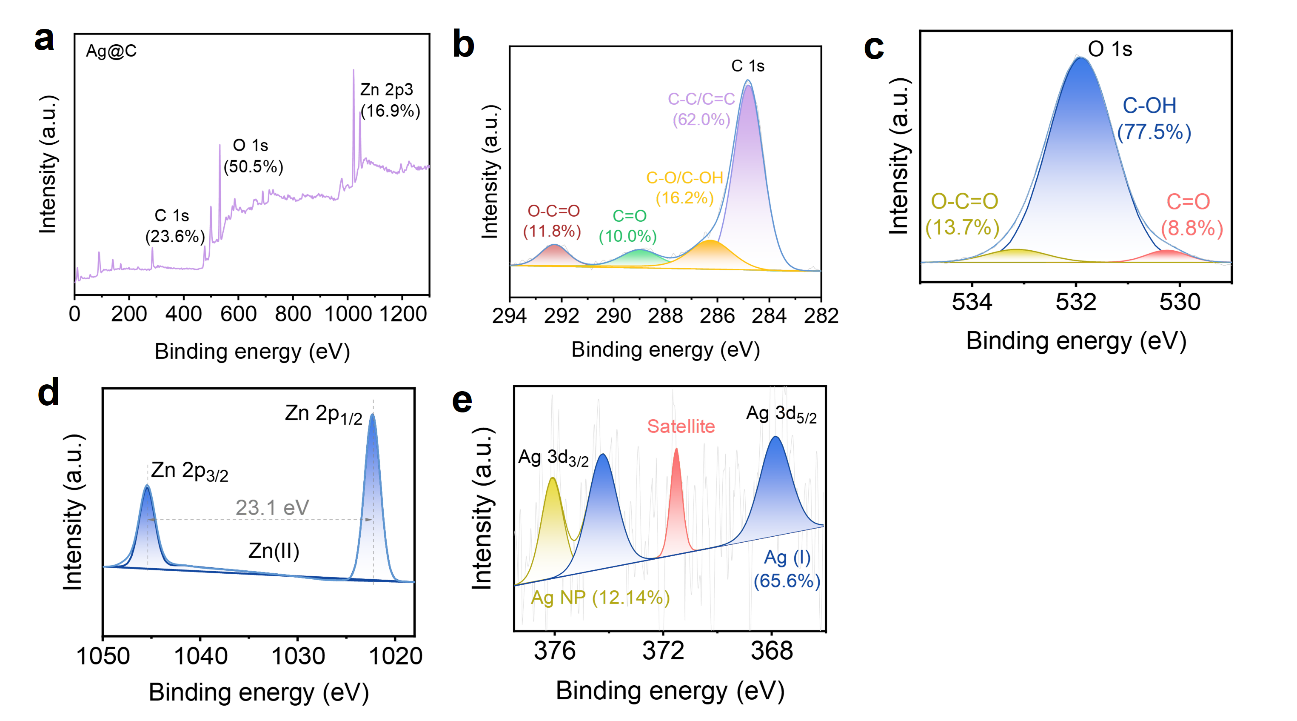


**Figure S28** The total XPS spectrum (a), high-resolution spectrum of C element (b), O element (c), Zn element (d), and Ag element on the surface of Ag@C electrode after 10,000 cycles.


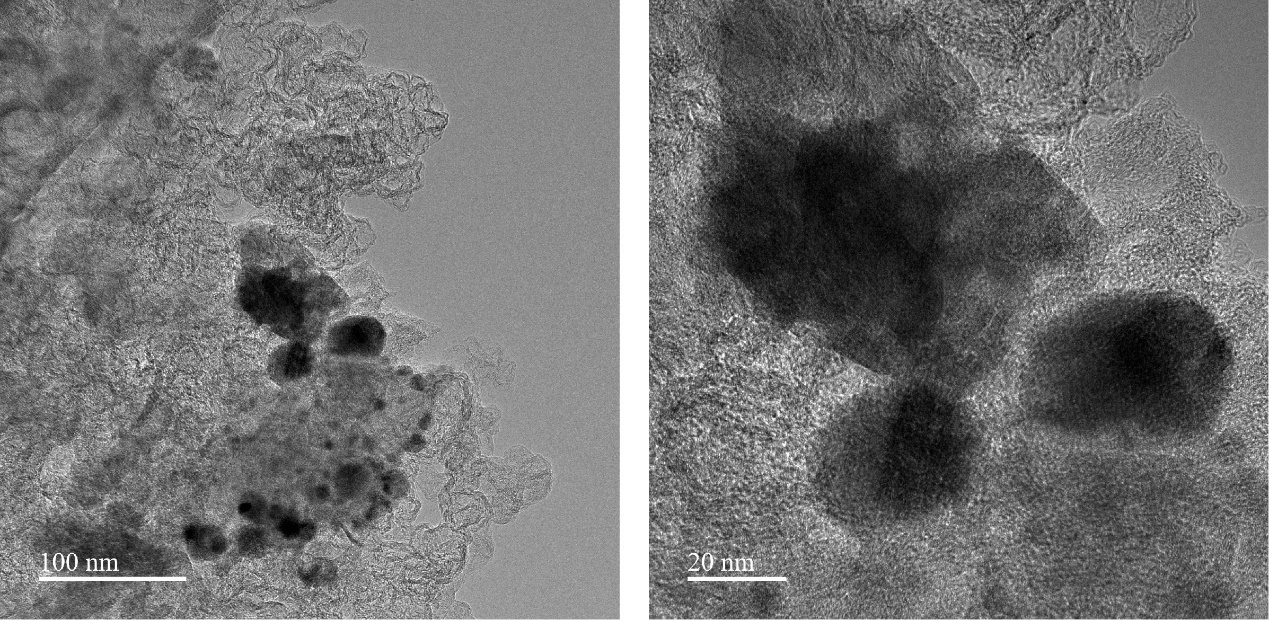


**Figure S29** The TEM and HR-TEM image of Ag@C sample after 10,000 cycles.

**References**

[1] Z. Liao, H. Y. Su, J. Cheng, G. T. Sun, L. Zhu, M. Q. Zhu, *Ind. Crops Prod.* **2021**, *171*, 113861.

[2] Z. W. Li, Y. F. An, S. Y. Dong, C. J. Chen, L. Y. Wu, Y. Sun, X. G. Zhang, *Energy Storage Mater.* **2020**, *31*, 252-266.

[3] X. Y. Jiang, Z. Z. Ouyang, Z. F. Zhang, C. Yang, X. Q. Li, Z. Dang, P. X. Wu, *Colloids Surface, A* **2018**, *547*, 64-72.

[4] F. X. Dong, L. Yan, X. H. Zhou, S. T. Huang, J. Y. Liang, W. X. Zhang, Z. W. Guo, P. R. Guo, W. Qian, L. J. Kong, W. Chu, Z. H. Diao, *J. Hazard. Mater.* **2021**, *416*, 125930.

[5] H. Zhang, H. Z. Wang, Z. H. Pan, Z. Wu, Y. D. Deng, J. P. Xie, J. Wang, X. P. Han, W. B. Hu, *Adv. Mater.* **2022**, *34*, 202206277.

[6] H. Aydın, Ü. Kurtan, B. Üstün, S. Koç, *Mater. Chem. Phys.* **2022**, *290*, 126392.

[7] J. X. Zhao, Z. F. Cong, J. Hu, H. Y. Lu, L. T. Wang, H. B. Wang, O. I. Malyi, X. Pu, Y. Y. Zhang, H. Y. Shao, Y. X. Tang, Z. L. Wang, *Nano Energy* **2022**, *93*, 106893.

[8] J. X. Zhao, H. Y. Li, C. W. Li, Q. C. Zhang, J. Sun, X. N. Wang, J. B. Guo, L. Y. Xie, J. X. Xie, B. He, Z. Y. Zhou, C. H. Lu, W. B. Lu, G. Zhu, Y. G. Yao, *Nano Energy* **2018**, *45*, 420-431.

[9] J. F. G. Kresse av, *Computational Materials Science* **1996**, *6*, 15-50.

[10] G. Kresse, J. Furthmuller, *Physical review. B, Condensed matter* **1996**, *54*, 11169-11186.

[11] P. E. Blochl, *Physical review. B, Condensed matter* **1994**, *50*, 17953-17979.

[12] J. P. Perdew, K. Burke, M. Ernzerhof, *Physical review letters* **1996**, *77*, 3865.

[13] H. J. Monkhorst, J. D. Pack, *Physical review B* **1976**, *13*, 5188.

[14] S. Grimme, *Journal of computational chemistry* **2006**, *27*, 1787-1799.
